# Self‐Activated Cascade‐Responsive Sorafenib and USP22 shRNA Co‐Delivery System for Synergetic Hepatocellular Carcinoma Therapy

**DOI:** 10.1002/advs.202003042

**Published:** 2021-01-15

**Authors:** Shengjun Xu, Sunbin Ling, Qiaonan Shan, Qianwei Ye, Qifan Zhan, Guangjiang Jiang, Jianyong Zhuo, Binhua Pan, Xue Wen, Tingting Feng, Haohao Lu, Xuyong Wei, Haiyang Xie, Shusen Zheng, Jiajia Xiang, Youqing Shen, Xiao Xu

**Affiliations:** ^1^ Department of Hepatobiliary and Pancreatic Surgery Affiliated Hangzhou First People's Hospital Zhejiang University School of Medicine Hangzhou Zhejiang 310006 China; ^2^ Department of Hepatobiliary and Pancreatic Surgery the First Affiliated Hospital School of Medicine Zhejiang University Hangzhou Zhejiang 310003 China; ^3^ NHC Key Lab of Combined Multi‐Organ Transplantation Hangzhou Zhejiang 310003 China; ^4^ Department of Pathology the First Affiliated Hospital School of Medicine Zhejiang University Hangzhou Zhejiang 310003 China; ^5^ Department of Abdominal Medical Oncology Zhejiang Cancer Hospital Hangzhou Zhejiang 310022 China; ^6^ Department of Polymer Science and Engineering Zhejiang University Hangzhou Zhejiang 310027 China; ^7^ Department of Hepatobiliary and Pancreatic Surgery Shulan (Hangzhou) Hospital Hangzhou Zhejiang 310000 China; ^8^ Center for Bionanoengineering and Key Laboratory of Biomass Chemical Engineering of Ministry of Education College of Chemical and Biological Engineering Zhejiang University Hangzhou Zhejiang 310027 China

**Keywords:** cascade‐responsive, co‐delivery, hepatocellular carcinoma, sorafenib, USP22 shRNA

## Abstract

Resistance to sorafenib severely hinders its effectiveness against hepatocellular carcinoma (HCC). Cancer stemness is closely connected with resistance to sorafenib. Methods for reversing the cancer stemness remains one of the largest concerns in research and the lack of such methods obstructs current HCC therapeutics. Ubiquitin‐specific protease 22 (USP22) is reported to play a pivotal role in HCC stemness and multidrug resistance (MDR). Herein, a galactose‐decorated lipopolyplex (Gal‐SLP) is developed as an HCC‐targeting self‐activated cascade‐responsive nanoplatform to co‐delivery sorafenib and USP22 shRNA (shUSP22) for synergetic HCC therapy. Sorafenib, entrapped in the Gal‐SLPs, induced a reactive oxygen species (ROS) cascade and triggered rapid shUSP22 release. Thus, Gal‐SLPs dramatically suppressed the expression of USP22. The downregulation of USP22 suppresses multidrug resistance‐associated protein 1 (MRP1) to induce intracellular sorafenib accumulation and hampers glycolysis of HCC cells. As a result, Gal‐SLPs efficiently inhibit the viability, proliferation, and colony formation of HCC cells. A sorafenib‐insensitive patient‐derived xenograft (PDX) model is established and adopted to evaluate in vivo antitumor effect of Gal‐SLPs. Gal‐SLPs exhibit potent antitumor efficiency and biosafety. Therefore, Gal‐SLPs are expected to have great potential in the clinical treatment of HCC.

## Graphical abstract

A galactose‐decorated lipopolyplex (Gal‐SLP) is developed as a hepatocellular carcinoma (HCC)‐targeting self‐activated cascade‐responsive nanoplatform to co‐deliver sorafenib and USP22 shRNA (shUSP22) for synergetic HCC therapy. Gal‐SLP exhibited a trio synergetic effect and achieved potent antitumor efficiency against a sorafenib‐insensitive patient‐derived xenograft.

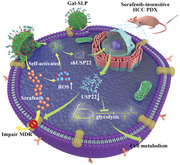

## 1 Introduction

Hepatocellular carcinoma (HCC), which has a continuously increasing incidence rate, remains a stumbling obstacle in increasing human life expectancy.^[^ ^1^ ^]^ Although appreciable progress in the treatment of HCC has occurred over the past few years, current therapeutic effects are still unsatisfactory, and the 5‐year survival rate varies from 14% to 18%.^[^ ^1^, ^2^ ^]^ Sorafenib, a kind of tyrosine kinase inhibitors, was approved by the FDA in 2007 for clinical applications as the first‐line drug against advanced HCC.^[^ ^3^ ^]^ Sorafenib inhibits cell proliferation and cancer angiogenesis by blocking the RAF/MEK/ERK pathway and tyrosine kinases of VEGFRs/PDGFRs.^[^ ^4^ ^]^ However, sorafenib often encounters clinical failure owing to drug resistance. Cancer stemness is closely associated with resistance to sorafenib and accounts for the clinical failure of current therapeutics against HCC. The glycolysis features of cancer cells, the tumor microenvironment and many other factors mediate HCC stemness.^[^ ^5^ ^]^ It is urgent to identify strategies for combating the current dilemma for HCC therapy.

Ubiquitin‐specific protease 22 (USP22), a member of the deubiquitination module of the SAGA chromatin‐modifying complex, plays an important role in cancer stemness via various mechanisms.^[^ ^6^ ^]^ An increasing number of studies have focused on the role of USP22 in malignant tumors.^[^ ^7^ ^]^ Our previous work revealed that USP22 was able to promote HCC stemness by a HIF1*α*/USP22 positive feedback loop and mediate multidrug resistance (MDR) by activating the SIRT1/AKT/MRP1 pathway. The expression of USP22 is associated tightly with the sensitivity of HCC cells to various chemotherapeutics (i.e., sorafenib, doxorubicin and fluorouracil).^[^ ^5^, ^8^ ^]^ Therefore, USP22‐specific gene therapy seems to be a promising method for reversing the cancer stemness and sensitizeing HCC cells to chemotherapeutics.

**Scheme 1:**
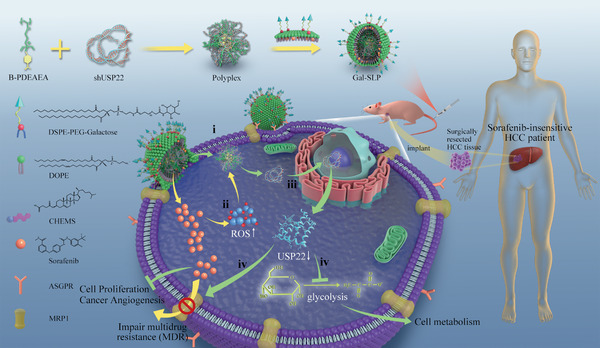
Schematic illustration of a self‐activated cascade‐responsive co‐delivery system (Gal‐SLP) for synergetic cancer therapy. i) B‐PDEAEA condenses USP22 shRNA (shUSP22) plasmids to form polyplexes. Polyplexes are further coated with a galactose‐decorated sorafenib‐loaded lipid layer to afford Gal‐SLPs. After intravenous injection, Gal‐SLPs accumulate in the tumor region, where the Gal ligands bind to the asialoglycoprotein receptors (ASGPRs) overexpressed on the cell membrane. The lipid layer fuses with the cell membrane and polyplexes are ejected into the cytosol. ii) Sorafenib is quickly released from Gal‐SLPs and induces elevated intracellular reactive oxygen species (ROS), which triggers dissociation of polyplexes and release of shUSP22. iii) The released shUSP22 enters the nucleus for transcription to specifically degrade USP22 mRNA and further suppress USP22 expression. iv) The downregulation of USP22 not only suppresses multidrug resistance‐associated protein 1 (MRP1), enhances the intracellular accumulation of sorafenib, and finally inhibits cell proliferation and cancer angiogenesis, but also inhibits glycolysis in hepatocellular carcinoma (HCC) cells, enhancing sorafenib chemosensitivity and impairing cell metabolism.

Tumor‐specific and efficient gene delivery remains an obstacle. An intelligent gene delivery nanoplatform, based on a reactive oxygen species (ROS)‐responsive charge‐reversal polymer (B‐PDEAEA), attracted increasing attention because the nanoplatform can avoid possible off‐target effects and achieve efficient gene transfection.^[^ ^9^ ^]^ However, rapid and efficient DNA release remains challenging due to the low concentration of ROS in cancer cells.^[^ ^10^ ^]^ Romain et al. mentioned that sorafenib induces ROS production more selectively in HCC cells than normal cells both in vitro and in vivo.^[^ ^11^ ^]^ The intracellular ROS elevation triggered by sorafenib was confirmed in our study through dihydroethidium (DHE) staining. Motivated by this phenomenon, sorafenib was adopted as an ROS‐generating agent to enhance the therapeutic effect of the current ROS‐responsive gene delivery system. In addition, USP22 was chosen as a therapeutic target for reversing cancer stemness and sensitizing HCC cells to sorafenib. Herein, we developed a self‐activated cascade‐responsive sorafenib and USP22 shRNA (shUSP22) co‐delivery system by simultaneously loading sorafenib and ROS‐responsive polyplexes in galactose‐decorated lipopolyplexes (Gal‐SLPs) (Scheme 1). Gal‐SLPs induced an exhaustive USP22 downregulation and exhibited marked in vitro and in vivo antitumor efficiency. In particular, Gal‐SLPs can induce a trio synergetic effect: i) sorafenib elevated intracellular levels of ROS, which oxidize B‐PDEAEA to trigger rapid shUSP22 release for efficient gene downregulation; ii) the downregulation of USP22 led to downregulation of multidrug resistance‐associated protein 1 (MRP1) and inhibition of glycolysis, dramatically impairing MDR and achieving higher intracellular sorafenib accumulation, thus generating an ROS‐responsive positive feedback loop; iii) the downregulation of USP22 suppressed the cell metabolism of cancer cells and further influenced cancer stemness. To further validate this strategy, antitumor efficiency was assessed, and a strong synergistic effect between sorafenib and shUSP22 gene therapy was observed in the present study.

## 2 Results

### 2.1 Construction of Polyplexes and Gal‐SLPs

**Figure 1:**
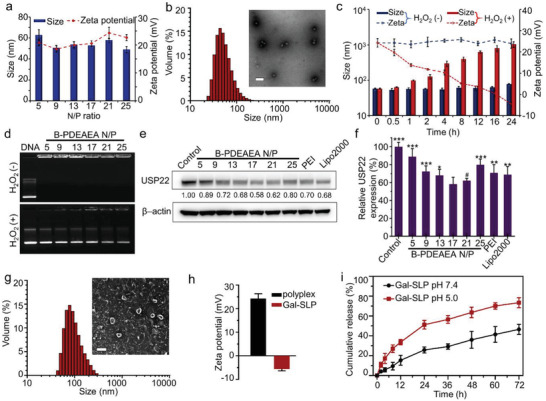
Characterization of polyplexes and Gal‐SLPs. a) Hydrodynamic diameters and zeta potentials of B‐PDEAEA/shUSP22 polyplexes at different N/P ratios. The data are presented as the mean ± SD (*n* = 3). b) Representative size distribution measured by dynamic light scattering (DLS) and transmission electron microscopy (TEM) images of B‐PDEAEA/shUSP22 polyplexes (N/P = 17). The scale bar is 200 nm. c) The change in the size and zeta potentials of polyplexes (N/P = 17) incubated with 200 µм H_2_O_2_ at 37 °C for different time intervals. The data are presented as the mean ± SD (*n* = 3). d) Gel retardation assay of polyplexes at different N/P ratios after an 1‐h incubation at 37 °C with or without H_2_O_2_. e,f) Representative Western blot analysis (e) of USP22 downregulation in Huh‐7 cells treated with polyplexes at different N/P ratios. Band intensities were semiquantified using IMAGE J software and normalized with *β*‐actin. Means are presented under the USP22 band (e) and data are presented as the mean ± SD (*n* = 3) (f). Polyethylenimine (PEI) and Lipofectamin 2000 (Lipo2000) were used as positive controls. Statistical differences and *p* value between two groups (versus N/P = 17) were shown as *p < 0.05, **p < 0.01, ***p < 0.001, and ^#^ *p* > 0.05. g) The size distribution measured by DLS and the morphology observed by TEM of Gal‐SLP (0.2 µmol lipid/µg DNA; N/P = 17). The scale bar is 200 nm. h) The zeta potential of the B‐PDEAEA/shUSP22 polyplex (N/P = 17) and Gal‐SLP. The data are presented as the mean ± SD (*n* = 3). i) In vitro sorafenib release from Gal‐SLPs in PBS containing 1% Tween 80 at pH 7.4 or 5.0. The data are presented as the mean ± SD (*n* = 3).

Polyplexes with various N/P ratios were prepared through electrostatic interactions between cationic B‐PDEAEA and anionic USP22 shRNA (shUSP22). B‐PDEAEA condensed shUSP22 and formed spherical polyplexes with uniform sizes of ≈50 nm and zeta potentials from +20 to +25 mV at N/P ratios ranging from **1**a–b After a 24‐h incubation with 200 µm H_2_O_2_, the zeta potential of polyplexes rapidly dropped from +24.3 to −4.6 mV and their size increased from 58.0 nm to ≈1 µm, which indicated a quick hydrolysis of the polymer and disintegration of polyplexes upon H_2_O_2_ (**Figure** **1**c). Accordingly, the ROS‐responsive charge‐reversal polyplexes released the DNA after a 1‐h incubation with 0.5 mm H_2_O_2_, as detected by gel electrophoresis (Figure 1d). Gene transfection efficiency and cytotoxicity assessments were performed to determine the optimum N/P ratio for further in vitro and in vivo experiments. B‐PDEAEA/shUSP22 polyplexes efficiently knocked down USP22 in both Huh‐7 and BEL‐7402 cells (Figure 1e–f, Figure S2, Supporting Information). In particular, ≈40% of USP22 was downregulated by B‐PDEAEA/shUSP22 polyplexes at an N/P ratio of 17 in Huh‐7 cells, which was significantly higher than that of conventional commercial vehicles (polyethylenimine [PEI] and Lipofectamin 2000 [Lipo2000]). The efficiency of the polyplexes was further evaluated using an EGFP‐shUSP22 plasmid. Likewise, polyplexes at an N/P ratio of 17 transfected optimally as determined by flow cytometry and fluorescence microscopy (Figures S3 and S4, Supporting Information). Moreover, polyplexes containing negative control (NC) plasmids showed negligible toxicity to Huh‐7 and BEL‐7402 cells among a wide range of N/P ratios (Figures S3a and S4a, Supporting Information).

To meet the requirement for intravenous administration, the positive charge of B‐PDEAEA/shUSP22 polyplexes should be shielded with polyanion or a lipid layer. Inspired by our previous report, a fusogenic lipid, consisting of 1,2‐dioleoyl‐sn‐glycero‐3‐phosphoethanolamine (DOPE), cholesteryl hemisuccinate (CHEMS) and distearoyl phosphoethanolamine–polyethylene glycol (DSPE‐PEG)‐galactose was used to enable the formation of lipopolyplex with stealth properties, HCC‐targeting ability and membrane fusion feature.^[^ ^9^, ^12^ ^]^ After decoration with a sorafenib‐loaded lipid layer, the zeta potential of Gal‐SLPs decreased to ‐5.6 ± 0.8 mV and the size increased to 95.6±5.2 nm (Figure 1g–h). Transmission electron microscopy (TEM) images of polyplexes and Gal‐SLPs (Figure 1b,g) also verified the successful decoration of the lipid layer. The drug loading content (DLC, %) and drug loading efficiency (DLE, %) of sorafenib in Gal‐SLPs were 3.6% and 74.5%, respectively. Further, sorafenib was released faster in the acidic environment than in the neutral environment, which indicated more favorable enhanced intratumoral sorafenib release (Figure 1i).

### 2.2 In Vitro Synergetic Effect of Sorafenib and shUSP22 Gene Therapy

**Figure 2:**
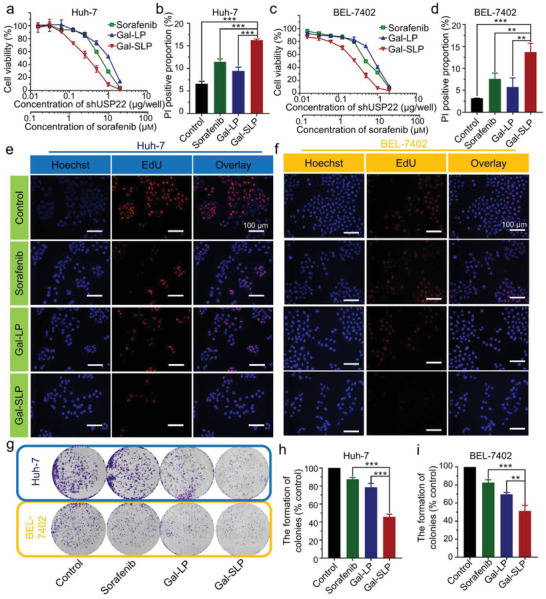
In vitro cytotoxicity assessment of sorafenib, Gal‐LP, and Gal‐SLP. a,c) Cytotoxicity of sorafenib, Gal‐LP and Gal‐SLP against Huh‐7 and BEL‐7402 cells after 48 h of treatment. The data are represented as the mean ± SD (*n* = 4). b,d) The apoptosis of Huh‐7 and BEL‐7402 cells was determined by propidium iodide (PI) staining. The cells were treated with sorafenib, Gal‐LPs or Gal‐SLPs for 48 h at a sorafenib concentration of 5 µм and shUSP22 concentration of 5 µg well^−1^. The data are represented as the mean ± SD (*n* = 3). e,f) Proliferation of Huh‐7 and BEL‐7402 cells treated with sorafenib, Gal‐LPs, and Gal‐SLPs at a sorafenib concentration of 5 µм and shUSP22 concentration of 8 µg dish^−1^ for 24 h. The cell nuclei were stained with Hoechst 33 342 (blue) and only proliferating cells were stained with EdU (red). Scale bars are 100 µm. g) Colony formation of Huh‐7 and BEL‐7402 cells treated with the indicated drugs for 14 days. h,i) Statistical analysis of the colony formation ability of Huh‐7 and BEL‐7402 cells upon different treatments. The data are represented as the mean ± SD (*n* = 3). *p < 0.05, **p < 0.01, ***p < 0.001, and ^#^ *p* > 0.05.

Cytotoxicity assessments in HCC cell lines, Huh‐7 and BEL‐7402, were performed using a Cell Counting Kit‐8 (CCK‐8) assay to investigate whether synergetic effects exist between sorafenib and shUSP22 gene therapy. Obviously, Gal‐SLPs exhibited much higher cytotoxicity against HCC cells (**Figure** **2**a,c). The half‐maximal inhibitory concentrations (IC_50_ values) of Gal‐SLPs against Huh‐7 cells were 2.8 µм (in terms of sorafenib dose) and 0.28 µg well^−1^ (in terms of shUSP22 dose), compared with 6.1 µм for sorafenib and 0.91 µg well^−1^ for galactose‐decorated liposomes without sorafenib (Gal‐LPs), a 2.2‐fold decrease and a 3.3‐fold decrease, respectively. The same phenomenon was observed in BEL‐7402 cells. Based on the the cell viability profiles in Figure 2a,c, combination index (CI_50_) of sorafenib and shUSP22 gene therapy was calculated via Equation (1) according to median‐effect analysis.^[^ ^13^ ^]^ CI_50_ of sorafenib and shUSP22 therapy against Huh‐7 and BEL‐7402 cells were 0.759 and 0.713, respectively, which indicated a synergetic effect of sorafenib and shUSP22 combinational therapy (Gal‐SLP) (Table 1). The cell death‐inducing capability of Gal‐SLPs was further studied by a propidium iodide (PI) staining assay (Figure 2b,d and Figure S5, Supporting Information). The percentage of dead cells upon Gal‐SLP treatment in Huh‐7 cells was 16.3 ± 0.25%, significantly higher than that of sorafenib (11.5±0.67%) and Gal‐LPs (9.5±0.82%) along, which was more obvious in BEL‐7402 cells. Gal‐SLPs led to 13.7 ± 2.0% BEL‐7402 cells death. In contrast, PI‐positive cell proportions in the sorafenib and Gal‐LP groups were 7.6 ± 1.3% and 5.7 ± 2.1%, respectively.

**Table 1:** IC_50_ and combination index (CI_50_) of sorafenib and shUSP22 gene therapy

	Huh‐7		BEL‐7402	
Samples	IC_50_	CI_50_	IC_50_	CI_50_
Sorafenib	0.6138	‐	0.5299	‐
Gal‐LP (shUSP22)	0.9134	‐	0.7538	‐
Gal‐SLP (sorafenib/shUSP22)	0.2792/0.2792	0.759	0.2219/0.2219	0.713

Sorafenib: µм; shUSP22: µg well^−1^.

The ability of Gal‐SLPs to inhibit cell proliferation and colony formation was evaluated in HCC cells via 5‐ethynyl‐2’‐deoxyuridine (EdU) incorporation and colony formation assays. EdU, a type of thymidine nucleoside analog, can be incorporated into DNA and label cells undergoing DNA replication. All the treatments inhibited cell proliferation (Figure 2e–f). It should be noted that Gal‐SLPs displayed an obviously better proliferation inhibition ability in contrast to the single treatment of sorafenib or Gal‐LPs. The clonogenic assay revealed that Gal‐SLPs induced a less colony formation and showed a long‐term synergistic antitumor effect (Figure 2g–i). All these data demonstrated that Gal‐SLPs were more efficient at decreasing the viability, proliferation and colony formation of HCC cells than sorafenib or Gal‐LPs alone.

### 2.3 Evaluation of Intracellular ROS and Gene Trafficking

**Figure 3:**
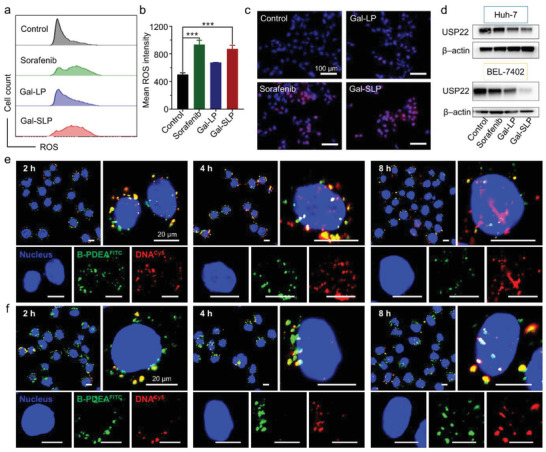
The mechanism of rapid and efficient intracellular shUSP22 release. a,b) Flow cytometry analysis and quantification of intracellular ROS generation in Huh‐7 cells incubated with sorafenib, Gal‐LPs or Gal‐SLPs at a sorafenib concentration of 5 µм and shUSP22 concentration of 8 µg dish^−1^ for 6 h. The data are represented as the mean ± SD (*n* = 3). c) Confocal microscopy images of intracellular ROS generation in Huh‐7 cells with the treatments mentioned above. Cell nuclei stained with Hoechst 33 342 are shown in blue, and ROS probed by DHE are shown in red. d) Western blot analysis of USP22 expression in Huh‐7 and BEL‐7402 cells. The cells were treated with sorafenib, Gal‐LPs or Gal‐SLPs at a sorafenib concentration of 5 µм and shUSP22 concentration of 8 µg dish^−1^ for 48 h. e,f) Intracellular dissociation of double labeled Gal‐SLPs (e) and Gal‐LPs (f) imaged by confocal laser scanning microscopy (CLSM). The cells were incubated with each lipopolyplex at a sorafenib concentration of 5 µм and a DNA concentration of 1 µg dish^−1^ for 2, 4 and 8 h. DNA labeled by Cy5 is shown in red, B‐PDEAEA labeled with fluorescein isothiocyanate (FITC) is shown in green and the nuclei stained with Hoechst 33 342 are shown in blue. All scale bars are 20 µm. *p < 0.05, **p < 0.01, ***p < 0.001 and ^#^ *p* > 0.05.

The ROS‐responsive charge‐reversal gene delivery system was utilized for shUSP22 delivery. The concentration of ROS in tumor cells could reach 0.5 nmol 10^4^ cells^−1^ h^−1^, which is higher than that in normal cells and avoids the therapeutic agents from off‐target effects.^[^ ^10c^ ^]^ However, intratumoral ROS are not high enough for the rapid and effective release of therapeutic agents and become an obstacle for the further development of ROS‐responsive delivery systems.^[^ ^14^ ^]^ Herein, we chose sorafenib, the first‐line treatment for unresectable HCC, as an ROS inducer. Sorafenib induces ROS production and ROS‐related cell death more selectively in HCC cells than normal cells both in vitro and in vivo, as indicated by previous studies.^[^ ^11^, ^15^ ^]^ The function of sorafenib as an inducer of ROS production was studied by flow cytometry and visualized by confocal laser scanning microscopy (CLSM). Dihydroethidium(DHE), which can be oxidized to fluorescent ethidium, was used as an ROS probe. Gal‐SLPs rapidly triggered ROS production after a 6‐h treatment, comparable to the results of free sorafenib (**Figure** **3**a,b). Moreover, strong red fluorescence was detected in the confocal images of Huh‐7 cells treated with sorafenib and Gal‐SLPs (Figure 3c), which suggested a high intracellular ROS concentration. In contrast, Huh‐7 cells in the control group had very weak fluorescence. These findings demonstrated that sorafenib released from Gal‐SLPs in tumor cells can induce a sharp elevation in intracellular ROS, which is expected to oxidize B‐PDEAEA and thus trigger a fast shUSP22 release for transcription.

We subsequently examined the USP22 downregulation efficiency of Gal‐SLPs using a Western blot assay. USP22 expression in Huh‐7 cells treated with Gal‐SLPs was much lower than that in Gal‐LP‐treated cells, and USP22 was almost knocked out in Gal‐SLP‐treated BEL‐7402 cells (Figure 3d), indicating that more shUSP22 was released from Gal‐SLPs as a result of sorafenib‐induced ROS elevation and was transcribed effectively to suppress USP22 expression. The intracellular dissociation and gene trafficking of Gal‐SLPs in Huh‐7 cells was investigated to reveal the mechanisms underlying the efficient USP22 downregulation, using doubly labeled lipopolyplexes consisting of Cy5‐labeled DNA (^Cy5^DNA, red) and FITC‐labeled B‐PDEAEA (^FITC^B‐PDEAEA, green) (Figure 3e,f). After a 2‐h incubation with Gal‐SLPs, some red dots were found in the cytosol, even in the nuclei, indicating fast DNA release from Gal‐SLPs and entry into nuclei for transcription, although more red and green fluorescence still overlapped (yellow dots). And with incubation expanding to 4 and 8 h, more red fluorescence was separated from the green fluorescence, and great red fluorescence was observed in the nuclear regions (Figure 3e). However, even after 8 h incubation with Gal‐LPs, few separated red dots were found in the nuclei, indicating slow polyplex dissociation and DNA release (Figure 3f). To further validate the role of sorafenib‐ or Gal‐SLP‐induced ROS in the quick release of shUSP22, *N*‐acetylcysteine (NAC) was utlized as a typical ROS scavenger. As shown in Figure S6a,b, Supporting Information, NAC efficiently lowered the concentration of Gal‐SLP‐induced ROS. More importantly, results of Western blot assay showed that ROS ablation by NAC inhibited the knockdown efficiency of USP22 by Gal‐SLP (Figure S6c, Supporting Information), which further supported that Gal‐SLP‐induced ROS participated in the quick release of shUSP22 for transcription. These results well proved that the charge‐reversal B‐PDEAEA/DNA polyplexes could dissociate quickly upon oxidation by the sorafenib‐induced elevation in ROS levels, and release DNA for quick nuclear entry and subsequent efficient transcription.

### 2.4 MRP1 Expression and Intracellular Sorafenib Accumulation

**Figure 4:**
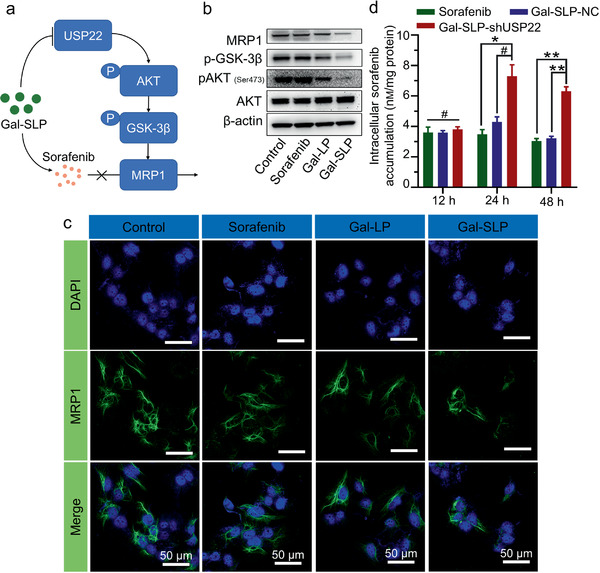
The downregulation of USP22 induced by Gal‐SLP efficiently suppresses MRP1 and leads to intracellular sorafenib accumulation. a) Illustration of the mechanisms by which Gal‐SLPs keeps cancer cells from pumping out sorafenib through MRP1. Gal‐SLPs could simultaneously release sorafenib and B‐PDEAEA/shUSP22 polyplexes. shUSP22 suppresses the expression of USP22 and further degrades MRP1 by blocking the AKT/GSK‐3*β* pathway, resulting in enhanced sorafenib accumulation in HCC cells. b) Western blot analysis of AKT, phosphorylated AKT (Ser473), phosphorylated GSK‐3*β* and MRP1 expression in Huh‐7 cells. The cells were incubated with sorafenib, Gal‐LPs or Gal‐SLPs for 48 h at a sorafenib concentration of 5 µм and shUSP22 concentration of 8 µg dish^−1^. c) Immunofluorescence staining of MRP1 in Huh‐7 cells treated with the indicated drugs for 48 h. The nuclei were stained with DAPI (blue) and MRP1 was labeled with CoraLite488 (green). All scale bars are 50 µm. d) Intracellular sorafenib accumulation in Huh‐7 cells treated with free sorafenib, Gal‐SLP‐NC (NC plasmid) and Gal‐SLP‐shUSP22 (shUSP22) for 12, 24, or 48 h. The data are represented as the mean ± SD (*n* = 3). *p < 0.05, **p < 0.01, ***p < 0.001 and ^#^ *p* > 0.05.

The expression of USP22 dramatically affects the expression of MRP1, which mediates MDR in numerous carcinomas by pumping anticancer agents out of cells.^[^ ^16^ ^]^ Downregulation of USP22 could inhibit the AKT/GSK‐3*β* pathway and further suppress the expression of MRP1 (**Figure** **4**a ^[^ ^8b^ ^]^ As illustrated in Figure 4b, MRP1, p‐AKT (Ser473) and phosphorylated GSK‐3*β* (p‐GSK‐3*β*) were downregulated in Huh‐7 cells treated with Gal‐LPs and Gal‐SLPs. Notably, the expressions of MRP1 and corresponding proteins in Gal‐SLP‐treated cells were much lower than that in Gal‐LP‐treated cells, which further supported the sorafenib‐induced intracellular ROS cascade and the ROS‐responsive gene transcription of Gal‐SLPs. Results of quantitative real time polymerase chain reaction (qRT‐PCR) assay were consistant with Western blot assay (Figure S7, Supporting Information). Gal‐SLPs efficiently downregulated the mRNA level of USP22 and MRP1. Moreover, the immunofluorescence staining was performed to visualize the expression of MRP1 (Figure 4c). Gal‐LPs and Gal‐SLPs significantly downregulated the expression of MRP1 in Huh 7 cells, particularly in Gal‐SLP‐treated cells, where MRP1 was hardly observed, whereas no obvious downregulation of MRP1 was observed in PBS‐ and sorafenib‐treated cells. Subsequently, intracellular sorafenib accumulation was quantified via HPLC analysis (Figure 4d). At 12 h, there were limited differences in sorafenib accumulation among cells treated with sorafenib, Gal‐SLP‐NC and Gal‐SLP‐shUSP22. However, with the treatment extending to 24 and 48 h, intracellular sorafenib accumulation in the Gal‐SLP‐shUSP22 treatment group was ≈1× higher than that in the sorafenib and Gal‐SLP‐NC groups. These results together provided strong evidence that Gal‐SLPs could induced an enhanced intracellular sorafenib accumulation through downregulating the expression of MRP1, thus hampering the efflux of sorafenib.

### 2.5 Glycolysis in Huh‐7 Cells with Different Treatments

**Figure 5:**
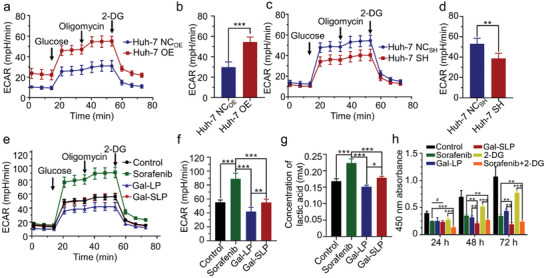
Gal‐SLP sensitizes Huh‐7 cells to sorafenib chemotherapy by suppressing glycolysis by downregulating of USP22. a,b) Extracellular acidification rate (ECAR) of Huh‐7 cells with USP22 NC or overexpression (OE) detected using the Seahorse system. The values of the seventh detection are represented as the mean ± SD in column graphs (*n* = 6). c,d) ECAR of Huh‐7 cells with USP22 NC or knockdown (SH). The values of the seventh detection are represented as the mean ± SD in column graphs (*n* = 6). e,f) ECAR of Huh‐7 cells treated with PBS, sorafenib, Gal‐LPs and Gal‐SLPs for 24 h. The values of the seventh detection are represented as the mean ± SD in column graphs (*n* = 6). g) Lactate production of Huh‐7 cells with the indicated treatments for 48 h. The data are represented as the mean ± SD (*n* = 3). h) Cytotoxicity of sorafenib, Gal‐LPs, Gal‐SLPs, 2‐DG and a combination of 2‐DG and sorafenib in Huh‐7 cells for 24, 48, and 72 h incubation. The concentrations of sorafenib, shUSP22 and 2‐DG was 5 µм, 0.5µg well^−1^ and 0.5 mm, respectively. The data are represented as the mean ± SD (n = 4). *p < 0.05, **p < 0.01, ***p < 0.001, and ^#^ *p* > 0.05.

We established USP22‐knockdown (SH) and USP22‐overexpressing (OE) HCC cells via lentiviral infection to explore the role of USP22 in sorafenib sensitivity. The results of Western blot assays verified the successful generation of Huh‐7 and BEL‐7402 cells with different expression levels of USP22 (Figure S8a,b, Supporting Information). The expression of USP22 was associated with the sensitivity to sorafenib (Figure S8c–e, Supporting Information). Compared with USP22‐NC_SH_ and USP22‐NC_OE_ HCC (Huh‐7 and BEL‐7402) cells, USP22‐SH HCC cells exhibited more sensitive to sorafenib, while USP22‐OE cells were more resistant to sorafenib. Meanwhile, glycolysis stress assay revealed that overexpression of USP22 increased the extracellular acidification rate (ECAR) of Huh‐7 cells (**Figure** **5**a), whereas downregulation of USP22 decreased the ECAR (Figure 5c,d). Thus, we speculated that the downregulation of USP22 by Gal‐SLPs could block the glycolysis and further suppress stemness features in HCC cells.

Accordingly, a glycolysis stress assay was performed in Huh‐7 cells with different treatments. Sorafenib greatly stimulated glycolysis of Huh‐7 cells, as evidenced by the greatly enhanced ECAR (Figure 5e,f) and lactate production (Figure 5g), which was one of the main reasons for the acquired drug resistance in clinical.^[^ ^17^ ^]^ Meanwhile, downregulation of USP22 by Gal‐LPs could reduce the ECAR and Gal‐SLPs offset the increase of ECAR triggered by sorafenib. Furthermore, a cytotoxicity assessment of sorafenib, Gal‐LPs, Gal‐SLPs, 2‐Deoxy‐d‐glucose (2‐DG) and a combination of 2‐DG and sorafenib was executed (Figure 5h). 2‐DG, a typical glycolysis inhibitor, sensitized HCC cells to sorafenib. It is worth noting that Gal‐SLPs inhibited more cell proliferation than sorafenib and Gal‐LPs alone. The inhibitory effect on glycolysis and the synergistic cell‐killing activity strongly indicated that Gal‐SLPs have an enhanced antitumor effect in a glycolysis‐inhibited manner.

### 2.6 Biodistribution and Pharmacokinetics Study of Gal‐SLPs

**Figure 6:**
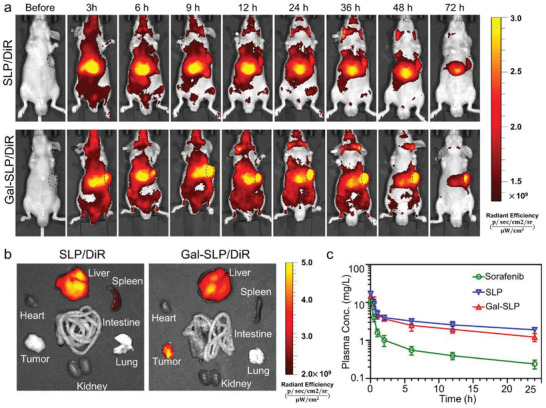
Biodistribution and pharmacokinetics of Gal‐SLP. HCC‐targeting gene delivery and long in vivo circulation of Gal‐SLP in the HCC patient‐derived xenograft (PDX) model. a) In vivo real‐time imaging of HCC PDX tumor‐bearing BALB/c nude mice after a single intravenous injection of DiR‐encapsulated Gal‐SLP (Gal‐SLP/DiR) and SLP (SLP/DiR) at a DiR dose of 2 µg per mouse. The dotted circle outlines the tumors. b) Ex vivo imaging of tumors and major organs (heart, liver, spleen, lung, kidney, and intestine) excised at 72 h post‐injection. c) Blood clearance profiles of sorafenib, SLP and Gal‐SLP after a single intravenous injection at a sorafenib dose of 5 mg kg^−1^ (*n* = 3). *p < 0.05, **p < 0.01, ***p < 0.001, and ^#^ *p* > 0.05.

Asialoglycoprotein receptors (ASGP‐R1 and R2), which are overexpressed in hepatocellular cancer cells, are favorable candidates for HCC‐targeting drug delivery.^[^ ^12^, ^18^ ^]^ Herein, galactose, a ligand for ASGPRs, was chosen to functionalize the lipopolyplexes to further enhance tumor accumulation.^[^ ^19^ ^]^ The in vivo real‐time distribution of lipopolyplexes was tracked on HCC PDX‐bearing BALB/c mice after a single intravenous injection of DiR‐loaded SLP (SLP/DiR) or Gal‐SLP (Gal‐SLP/DiR). As exhibited in **Figure** **6**a, a strong fluorescence derived from DiR was observed throughout the whole body and gradually accumulated in the tumor regions in both the SLP/DiR and Gal‐SLP/DiR groups. Notably, the fluorescence signals of SLP/DiR and Gal‐SLP/DiR decayed slowly, which indicated that lipopolyplexes have a long in vivo circulation period. Compared with SLP/DiR, obviously enhanced fluorescence signal was detected in the tumor region in the Gal‐SLP/DiR group 9 h post‐injection and the high contrast between the tumor area and surrounding tissues sustained throughout the following experimental period. At 72 h post‐injection, tumors and organs were excised from the mice and ex vivo imaging was performed (Figure 6b). The tumor accumulation of DiR in the Gal‐SLP/DiR group was clearly higher than that in the SLP/DiR group, demonstrating an effective tumor‐targeting capability of galactose‐functionalized Gal‐SLP.

An in vivo pharmacokinetic study was conducted to investigate drug retention in the blood circulation. Blood was collected from ICR mice after a single intravenous injection of sorafenib, SLP and Gal‐SLP at a sorafenib dose of 5 mg kg^−1^ and sorafenib content in the blood was quantified by HPLC analysis. The concentration‐time profile and pharmacokinetic parameters of sorafenib are presented in Figure 6c and Table 2. Compared with the rapid clearance of sorafenib, both SLPs and Gal‐SLPs exhibited substantially extended circulation in vivo. The area under the curve (AUC_0‐t_) values of SLP and Gal‐SLP were 71.4 ± 14.6 and 58.7 ± 8.98 µg × h mL^−1^, respectively, while the AUC_0‐_ *_t_* value of free sorafenib was relatively low (16.3 ± 3.48 µg × h mL^−1^). These data supported that lipopolyplexes helped prolong the blood circulation of sorafenib, which was in accordance with the in vitro sorafenib release kinetics and biodistribution study.

**Table 2:** Pharmacokinetic parameters of sorafenib, SLP, and Gal‐SLP at a sorafenib injection concentration of 5 mg kg^−1^

	Drug formulation		
PK parameters	Sorafenib	SLP	Gal‐SLP
*t*_1/2*α*_ [h]	0.28 ± 0.01	0.31 ± 0.09	0.44 ± 0.16
*t*_1/2*β* _[h]	13.0 ± 1.43	21.7 ± 3.06	18.5 ± 1.23
CL	0.16 ± 0.04	0.03 ± 0.01	0.03 ± 0.01
AUC_(0‐_ *_t_*_) _[µg × h mL^−1^]	16.3 ± 3.48	71.4 ± 14.6	58.7 ± 8.98

### 2.7 In Vivo Antitumor Effects on Sorafenib‐Insensitive HCC Patient‐Derived Xenografts

**Figure 7:**
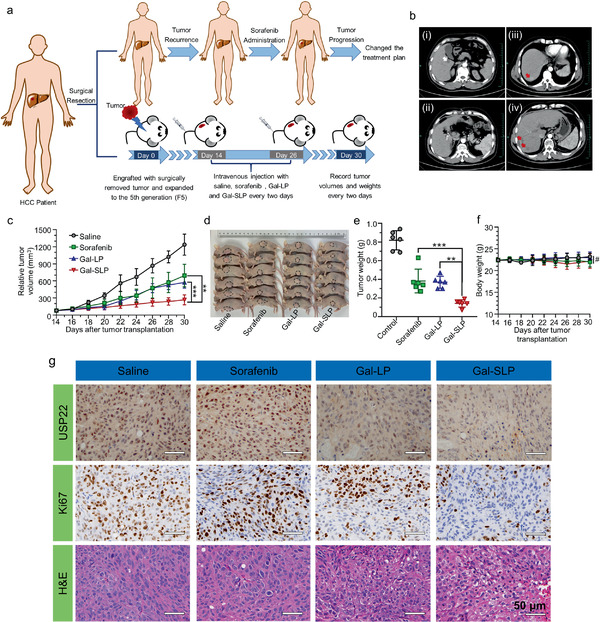
The antitumor activity of Gal‐SLP in a sorafenib‐insensitive HCC PDX model. a) Schematic illustration of the establishment of a sorafenib‐insensitive HCC PDX model and the experimental timeline and dosing regimens in the Patient 25‐derived (P25) mice. b) Representative CT scan images of HCC Patient 25 (P25) at different times. i) Diagnosis of HCC before surgical resection. ii) Postoperative changes in the liver. iii) One month after surgery and tumor recurrence. iv) Half a year after surgery and tumor progression. The white arrow indicates the tumor region, the black arrow indicates the titanium clip after the surgical resection, and the red arrows indicate the tumor recurrence and progression. c) The tumor growth curves after intravenous injection of saline, sorafenib (formulated in Cremophor EL/ethanol, v/v = 1:1), Gal‐LP and Gal‐SLP (*n* = 6, sorafenib dose, 5 mg kg^−1^; shUSP22 dose, 0.5 mg kg^−1^) according to a q2 × 6 regimen. d) Images of the whole‐body of the mice at the experimental endpoint. e) The average tumor weight of the excised tumor in each group at the experimental endpoint (*n* = 6). f) Body weight variation in the tumor‐bearing mice during the experimental period (*n* = 6). g) Representative images of IHC staining (USP22 and Ki67) and H&E staining of tumors on day 30. *p < 0.05, **p < 0.01, ***p < 0.001, and ^#^ *p* > 0.05.

The patient‐derived xenografts (PDX) applied in this study was originated from a patient, who didn't have any chemotherapeutic or locoregional treatments before the surgery. The patient was treated with transarterial chemoembolization (TACE) thrice, radiofrequency ablation (RFA) once and long‐term oral administration of sorafenib immediately after HCC recurrence and reduced the sorafenib dosage to half due to the severe hand‐foot skin reaction. The time to progression (TTP) of this patient was 5.5 months, less than the median TTP (6.3 months for the combination of TACE and sorafenib) reported previously, which indicated that the patient responded poorly to the combinational therapy.^[^ ^20^ ^]^ Finally, the patient changed treatment plans due to rapid tumor progression. The illustration of establishing sorafenib‐insensitive HCC PDX models and tumor progression of Patient‐25 was shown in **Figure** **7**a,b.

We explored whether our strategy to co‐delivery sorafenib and shUSP22 via Gal‐SLP was promising for enhancing curative effect on the sorafenib‐insensitive HCC PDX model. Saline, sorafenib, Gal‐LP and Gal‐SLP were intravenously injected every other day for 6 times at a sorafenib dose of 5 mg kg^−1^ and shUSP22 dose of 0.5 mg kg^−1^. Compared with saline, all sorafenib, Gal‐LP and Gal‐SLP led to significant tumor growth inhibition, although a single treatment of sorafenib or Gal‐LP showed very limited anticancer efficacy with a fast tumor rebound during treatment‐free period (Figure 7c,d, Figure S9, Supporting Information). However, Gal‐SLPs exhibited much better antitumor efficiency, strongly suppressing the tumor growth during the whole experimental period, resulting in a tumor‐growth inhibition rate (TIR %) of 82.7 ± 4.9% in terms of tumor weight at the end of experiment, significantly higher than that of sorafenib (53.4 ± 15.5%) and Gal‐LPs (55.0 ± 7.8%) (Figure 7d,e, Figure S10, Supporting Information). Histological analysis via hematoxylin and eosin (H&E) staining of the tumors tissue sections showed numerous apoptotic cells with extensive vacuolization, severe nucleus shrinkage and decreased cellularity in the Gal‐SLP‐treated group, compared with the tightly‐packed tumor cells in the saline group and much reduced apoptotic characteristics in sorafenib and Gal‐LP groups, supporting the strong tumor inhibition activity of Gal‐SLP (Figure 7g). The results of immunohistochemistry (IHC) staining revealed that Gal‐SLPs efficiently suppressed the expression of Ki67, a marker for tumor proliferation, further confirming the excellent antitumor capability of Gal‐SLP. Besides, Gal‐SLPs downregulated in vivo USP22 expression to a much greater extent than Gal‐LPs (Figure 7g), which was well correlated with the in vitro Western blot analysis, convincingly proving the self‐activated cascade‐responsive process and synergy of sorafenib and shUSP22.

For biosafety evaluation, organs were excised from nude mice on day 30 and analyzed by H&E staining. There was no notable damage in any of the treatment groups (Figure S11, Supporting Information). The administration of sorafenib, Gal‐LPs or Gal‐SLPs did not influence the weights of mice (Figure 7f). In vivo safety evaluation, performed in the healthy ICR mice, revealed no renal or liver damage (Figure S12, Supporting Information). These data supported the excellent safety profile of Gal‐SLPs.

## 3 Discussion and Conclusion

Sorafenib is the first FDA‐approved MKI for unresectable HCC and remains the first‐line therapy. However, only ≈40% of HCC patients can benefit from sorafenib owing to cancer stemness and other reasons.^[^ ^1^, ^2^, ^3^ ^]^ Methods for reversing cancer stemness and sensitizing HCC patients to sorafenib remain some of the largest concerns for improving the prognosis of HCC patients. USP22, as a cancer stem cell (CSC) marker, plays a critical role in cancer stemness and has drawn more and more attention in the recent researches.^[^ ^5^, ^6^, ^7^, ^21^ ^]^ USP22‐targeted gene therapy seems to be a promising way out of the current dilemma in HCC therapy. To this end, we developed a self‐activated cascade‐responsive sorafenib and shUSP22 co‐delivery system (Gal‐SLP) for synergetic HCC therapeutics.

Characterizations of the sorafenib/shUSP22 co‐delivery nanoplatform showed that Gal‐SLPs, with an average diameter of ≈100 nm, exhibited excellent ROS responsiveness and high gene transfection efficiency. Sorafenib, entrapped in the Gal‐SLPs, displayed sustained and acid‐favored release kinetics. Once Gal‐SLPs entered HCC cells, sorafenib was released immediately and worked as an ROS inducer to trigger the rise of intracellular ROS to further induce a rapid release of shUSP22 from polyplexes. Only a few Huh‐7 and BEL‐7402 cells treated with Gal‐SLPs expressed USP22. Accordingly, a series of in vitro cytotoxicity experiments demonstrated that Gal‐SLPs were more efficient than sorafenib and Gal‐LPs suppressing the growth, proliferation and colony formation of HCC cells. Additionally, cloaking the surface with a galactose‐decorated lipid layer not only extended the blood circulation of Gal‐SLPs, but also enabled the nanoplatform to specifically co‐deliver sorafenib and shUSP22 to the tumor region.

In our study, USP22 shRNA gene therapy was utilized to reverse HCC stemness and sensitize HCC cells to sorafenib. On the one hand, our previous work has indicated that USP22 is closely associated with HCC stemness through the modulation of glycolysis.^[^ ^5a^ ^]^ In this study, we observed that the downregulation of USP22 by lentiviral infection or shUSP22 gene therapy (Gal‐LP) was able to suppress glycolysis of Huh‐7 cells. However, glycolysis was activated by sorafenib administration and this phenomenon was believed to connect tightly with cancer stemness and to further mediate resistance to sorafenib.^[^ ^22^ ^]^ Gal‐SLPs inhibited the potential activation of glycolysis by sorafenib and sensitized HCC cells to sorafenib, which was familiar with the sorafenib sensitabling effect of 2‐DG. On the other hand, USP22 was proven to participate in MDR by modulating MRP1 through the AKT/GSK‐3*β* pathway.^[^ ^8b^ ^]^ Our study provided strong evidence that the downregulation of USP22 by Gal‐SLPs suppressed the expression of MRP1 and caused high intracellular sorafenib accumulation. Sorafenib accumulation not only suppressed the cell proliferation and cancer angiogenesis, but also generated an ROS‐responsive positive feedback loop to trigger more shUSP22 release and sorafenib accumulation. Our study showed that Gal‐SLP‐shUSP22 induced higher intracellular sorafenib accumulation in Huh‐7 cells after 24 and 48 h incubation. Furthermore, recent studies indicated USP22 played an important role in modulating of PD‐L1 expression in cancer cells and maintaining *T*_reg_ cell suppression function,^[^ ^7^, ^21^, ^23^ ^]^ which suggested that USP22‐targeted gene therapy was not only essential to sensitive cancer cells to sorafenib, but also meaningful for modulating the cancer immune microenvironment and enhancing the efficiency of anticancer immunotherapy. This is a focus area of our future work.

Identification of the most effective, least toxic, and cost‐effective treatment plan for cancer patients is still the main problem faced by clinicians. PDX models have been widely adopted as a powerful tool in the evaluation of drug efficiency and offer assistance in making individual precision medical plans because PDX models can retain the unique features (such as gene patterns, histological properties and responses to drug treatment) of tumors in human patients.^[^ ^24^ ^]^ In this study, a PDX model was established as an avatar of a sorafenib‐insensitive HCC patient for in vivo antitumor evaluation. Once injected into mice, more Gal‐SLPs accumulated in the tumor site owing to the EPR effect and active targeting. And contributed by self‐activated cascade‐responsive design, shUSP22 encapsulated in Gal‐SLPs was apt to release more rapidly than that in Gal‐LP. As a result, Gal‐SLP dramatically suppressed the USP22 expression in the tumor and lead a great proportion of apoptotic cells. Compared with sorafenib or Gal‐LP single treatment, Gal‐SLP was more potent in this HCC PDX model to inhibit the tumor growth. The excellent in vitro and in vivo antitumor efficiency of Gal‐SLPs suggested that the patient from whom, the PDX originated, might benefit from this sorafenib and shUSP22 combination therapy.

In summary, we developed a self‐activated cascade‐responsive sorafenib and shUSP22 co‐delivery system (Gal‐SLP) for synergetic HCC therapeutics. Gal‐SLPs exhibited potent antitumor efficiency via a trio synergetic effect and are expected to be promising for HCC therapy.

## 4 Experimental Section

##### Preparation and Characterization of Polyplexes with Different N/P Ratios

HEPES buffer solution (10 mm, pH = 7.4) was used to dilute plasmid DNA to a concentration of 40 µg mL^−1^. According to the preset N/P ratios (N/P = 5, 9, 13, 17, 21, 25), B‐PDEAEA was dissolved in HEPES buffer solution (10 mm, pH = 7.4) at various concentrations. And then, an equal volume of DNA solution was added to the B‐PDEAEA solution. The mixture was immediately vortexed for 10 s, followed by incubating statically for 30 min. Sizes and zeta potentials of polyplexes were measured by dynamic light scattering (DLS) (Malvern, UK).

##### USP22 Knockdown Efficiency and Cytotoxicity Assessments of Polyplexes

For USP22 knockdown efficiency assessment, 150 000 HCC cells in 6‐well plates were treated with polyplexes containing shUSP22 plasmids at a shUSP22 concentration of 8 µg well^−1^ for 48 h and then harvested. Lipo2000 and branched polyethylenimine (PEI, 25 kDa) were used as positive controls. Western blot assay was performed as described previously.^[^ ^5^, ^8^ ^]^ Bands were incubated with primary antibody dilution overnight, followed by incubation with secondary antibody dilution for 2 h. Finally, bands were visualized with ECL detection reagents (Fude Biological Tech.). All antibodies were diluted according to manufacturers’ recommendations and supplied in Table S1, Supporting Information.

Cytotoxicity of polyplexes was determined by CCK‐8 assay (Dojindo). 3000 HCC cells were seeded into 96‐well plates and incubated overnight. Polyplex contained negative control (NC) plasmids with different N/P ratios was added into medium at a DNA concentration of 0.5 µg well^−1^ and incubation was continued for an additional 48 h. When the incubation was finished, the medium was replaced with 110 µL fresh medium containing 10 µL CCK‐8 solution. After a further incubation for 2 h, the absorbance of 450 nm of each well was measured via microplate reader (ELx800, Bio Tek).

##### DNA Encapsulation Efficiency and ROS‐Responsiveness Assessment of Polyplexes

DNA encapsulation efficiency of polyplexes with various N/P ratios was evaluated by gel retardation assay. The polyplexes were loaded into a 0.8% agarose gel and electrophoresed in TBE buffer at 120 V for 45 min using GT Mini‐Gel Casting System (BIO‐RAD). There existed Gel Red (Biotium) in the agarose gel for DNA detection. For ROS‐responsiveness assessment, polyplexes were treated with 0.5 mm H_2_O_2_ at 37 °C for 1 h, followed by electrophoresis.

The changes of sizes and zeta potentials of polyplexes were measured using DLS. Polyplexes (N/P = 17) were incubated with 200 µм H_2_O_2_ at 37 °C. At predetermined timepoints, 200 µL of sample was collected for size and zeta potential measurements.

##### Preparation and Characterization of Galactose‐Decorated Sorafenib‐Loaded Lipopolyplexes (Gal‐SLPs, N/P = 17)

Galactose‐decorated sorafenib‐loaded lipopolyplex was prepared by the thin‐film hydration method.^[^ ^9^ ^]^ The molar ratio of sorafenib/DOPE/CHEMS/ DSPE‐PEG2000‐galactose was fixed at 1.0:6.9:1.8:1.2. Sorafenib (0.23 mg), DOPE (2.53 mg), CHEMS (0.43 mg), and DSPE‐PEG2000‐galactose (1.84 mg) were dissolved in 2 mL chloroform and the organic solvent was removed by rotary evaporation to afford a thin lipid film. The film was hydrated overnight in 1 mL HEPES (10 mm, pH 7.4) at room temperature with stirring, followed by ultrasonication for 10 min in ice bath. The lipopolyplex solution was then stored at 4 °C for 2 h and filtered through a 0.22 µm nylon filter to remove non‐encapsulated drug aggregates. Gal‐SLPs was prepared as previously published.^[^ ^9^, ^25^ ^]^ The previous lipopolyplex solution (500 µL) was added to the polyplexes solution (N/P = 17, 500 µL, 10 µg DNA) and incubated overnight at room temperature. The size and zeta potential of Gal‐SLP were assessed using DLS and the morphology of Gal‐SLP was observed and imaged by TEM (TECNAL 10, Philips) after negative staining. For the determination of drug loading content (DLC) and drug loading efficiency (DLE), Gal‐SLP was lyophilized and redissolved in acetonitrile for HPLC analysis. Release process of sorafenib from Gal‐SLP was explored via the dialysis method. Briefly, Gal‐SLP solution (2 mL, containing 0.5 mg sorafenib) was sealed in a dialysis bag (MWCO = 3500 Da) and dialyzed against 60 mL of PBS containing 1% tween 80 at different pH (pH = 5.0 or 7.4). At predetermined time intervals, 100 µL of the dialysate was withdrawn for HPLC analysis.

##### In Vitro Cytotoxicity Assay

In vitro cytotoxicity assay was determined by CCK‐8 assay and PI staining assay. HCC cells were seeded into 96‐well plates at a density of 3000 cell/well and cultured overnight before treatment. Then, sorafenib, Gal‐LP and Gal‐SLP were added into medium at virous sorafenib and shUSP22 concentrations. After incubation for 48 h, cell viability was assessed by CCK‐8 assay as mentioned above. For PI staining, 100 000 HCC cells (Huh‐7 and BEL‐7402) were plated into 6‐well plates. Sorafenib, Gal‐LP and Gal‐SLP were added at a sorafenib concentration of 5 µм and shUSP22 concentration of 5 µg well^−1^. After treatment for 36 h, cells were harvested and stained with 5 µL PI solution (1 µg mL^−1^, KeyGen) for 15 min. PI‐positive cells proportion was quantitatively investigated by flow cytometer (BD FACSCanto II).

##### Synergy of Sorafenib and shUSP22 Gene Therapy

Based on the cell viability profiles in Figure 2a,c, combination index (CI_50_) was calculated via Equation (1) according to median‐effect analysis.^[^ ^13^ ^]^

(1) $$CI_{50}=\frac{IC_{\mathrm{sorafenib}\;\mathrm{in}\;\mathrm{Gal}-\mathrm{SLPs}}}{IC_{\mathrm{single}\;\mathrm{sorafenib}}}+\frac{IC_{\mathrm{shUSP}22\;\mathrm{in}\;\mathrm{Gal}-\mathrm{SLPs}}}{IC_{\mathrm{shUSP}22\;\mathrm{in}\;\mathrm{Gal}-\mathrm{LPs}}}$$

where CI_50_ represents the combination index of sorafenib and shUSP22 gene therapy in the combination (Gal‐SLP) that inhibits a half of cells; IC_sorafenib in Gal‐SLPs_ and IC_shUSP22 in Gal‐SLPs_ are the concentration of sorafenib and shUSP22 in Gal‐SLPs that inhibits 50% of cells; IC_single sorafenib_ and IC_shUSP22 in Gal‐LPs_ are the concentration of sorafenib and shUSP22 (Gal‐LPs), respectively, that inhibits 50% of cells individually. The classifications of synergy are additive (CI_50_ = 1), synergistic (CI_50_<1), or antagonistic (CI_50_>1). IC_50_ value was calculated using GraphPad Prism software.

##### Western Blot Analysis

To detect the USP22 and its related protein (MRP1, p‐GSK‐3*β*, p‐AKT) expression levels, 200 000 HCC cells were plated into Petri dishes. After treatment with sorafenib, Gal‐LPs and Gal‐SLPs for 48 h at a sorafenib concentration of 5 µм and shUSP22 concentration of 8 µg dish^−1^ respectively, cells were harvested, followed by Western blot assay as mentioned above. All antibodies were diluted according to manufacturers’ recommendations and supplied in Table S1, Supporting Information.

##### Cell Proliferation Assay

Cell proliferation was investigated by staining cells with Click‐iT EdU Imaging Kit (Invitrogen). EdU staining was carried out according to manufacturer's protocols. In a nutshell, 20 000 HCC cells were seeded in glass bottom petri dishes. After the same treatment as mentioned in the Western blot analysis for 24 h, an equal volume of 20 µм EdU solution was added into medium. Cells were incubated for further 30 min, followed by cell fixation with 4% paraformaldehyde (PFA) fixation solution and permeabilization with 0.5% Triton X‐100 solution. Alexa Fluor 488 azides were added and incubated for additional 30 min at room temperature and cell nuclei were stained with Hoechst 33 342 (Thermo) for 30 min. The variations of fluorescence were detected using a fluorescence microscopy.

##### Colony Formation Assay

HCC cells were seeded into 6‐well plates at a density of 2000 cell well^−1^ and cultured for 12 h before treatment. Cells were treated with sorafenib, Gal‐LPs and Gal‐SLPs at a sorafenib concentration of 0.5 µм and shUSP22 concentration of 5 µg well^−1^. Every third day, the medium was replaced with fresh medium containing the same concentrations of sorafenib, Gal‐LPs and Gal‐SLPs. After 2‐week treatment, cells were stained with 0.1% crystal violet solution at room temperature for 15 min and washed lightly with PBS solution for three times. Colonies were photographed for further colony formation counting.

##### Intracellular ROS Evaluation

Huh‐7 cells were plated into 6‐well plates at a density of 100 000 cell well^−1^ and cultured overnight. Huh‐7 cells were exposed to sorafenib, Gal‐LPs and Gal‐SLPs for 6 h at a sorafenib concentration of 0.5 µм and shUSP22 concentration of 8 µg well^−1^. And then the medium was replaced with serum‐free medium containing 10 µм dihydroethidium (DHE). After incubation for half an hour, cells were collected, washed and suspended three times with PBS solution. The intracellular ROS intensity was quantitatively assessed using a flow cytometer.

For visualization of intracellular ROS elevation, 20 000 Huh‐7 cells were cultured in glass bottom petri dishes for 12 h before treatment. After treated with PBS, sorafenib, Gal‐LPs and Gal‐SLPs for 6 h, the medium was replaced with serum‐free medium containing 10 µм DHE. After staining for 30 min, cells were washed with PBS three times and fixation with 4% PFA fix solution. And then nuclei were stained with Hoechst 33 342. Hence, cell images were acquired on a confocal microscope with constant imaging parameters for different groups.

##### Cellular Uptake and Intracellular Trafficking Study

Huh‐7 cells were plated onto glass bottom petri dishes at a density of 20 000 cells per dish in 1.5 mL medium and cultured for 24 h. The medium was replaced with 1 mL fresh medium containing Gal‐LPs or Gal‐SLPs at a DNA concentration of 1 µg dish^−1^. After timed incubation, the cells were further stained with Hoechst 33 342 for 20 min to label the nuclei. Cells were then washed thrice with PBS and fixed with 4% PFA fix solution before CLSM observation.

##### MRP1 Immunofluorescence Assay

15 000 Huh‐7 cells were seeded in glass bottom petri dishes and treated with sorafenib, Gal‐LPs and Gal‐SLPs at a sorafenib concentration of 5 µм and shUSP22 concentration of 2.5 µg dish^−1^ for 48 h. MRP1 immunofluorescence assay was performed as manufacturers recommended. Briefly, the cells were fixed in methanol, blocked and permeabilized using 1% BSA solution for 1 h at room temperature. The cells were then incubated with the diluted MRP1 antibody for 16 h at 4 °C. Solution was decanted and cells were washed three times with PBS. The cells were further incubated with diluted CoraLite488—conjugated second antibody for 1 h at room temperature and washed with PBS three times. At last, cells were stained with Hoechst 33 342 and observed in CLSM (Nikon A1).

##### Intracellular Sorafenib Accumulation Evaluation

100 000 Huh‐7 cells were seeded in 10 cm dishes and treated with sorafenib, Gal‐SLP‐NC (containing NC plasmid) and Gal‐SLP‐shUSP22 (containing shUSP22 plasmid) at a sorafenib concentration of 5 µм and a plasmid concentration of 15 µg dish^−1^ for 12, 24, and 48 h. When the incubation was finished, the medium was discarded and cells were rinsed with PBS. The cells were harvested and lysed with 100 µL RIPA lysis buffer with ice bath for 30 min. The lysate was centrifuge at 12 000 rpm in 4 °C for 10 min. Protein, contained in the supernatant, was quantified via BCA assay (Thermo). The resident lysate was further lyophilized and dissolved in acetonitrile for HPLC analysis.

##### Glycolysis Stress Assay

Extracellular acidification rates (ECAR), which directly reflect glycolytic rates, were monitored using Seahorse XFe96 Analyzer (Agilent) as manufacturers recommended. ECAR of Huh‐7 USP22‐NC_OE_, OE, NC_SH_, and SH cells, which were mentioned in the Supporting Information, were measured. Meanwhile, ECAR of Huh‐7 cells, exposed to different treatments, were further assessed. Huh‐7 cells, cultured in Petri dishes, were treated as mentioned in the Western blot analysis for 24 h before seeded into 96‐well microplates that were supplied by Agilent. All ECAR data were analyzed and presented using Seahorse Wave 2.6 and GraphPad Prism 8 according to manufacturers’ instructions.

##### Lactate Production Assay

Lactate production was monitored by Lactic Acid LD Kit (KeyGen). 50 000 Huh‐7 cells were cultured in Petri dishes for 12 h before treatment. The treatment was as same as mentioned in the Western blot analysis for 24 h. Finally, the medium was harvested and concentrations of lactate were measured in line with kit manual.

##### Cytotoxicity Assessment of Sorafenib, Gal‐LP, Gal‐SLP, 2‐DG, and a Combination of 2‐DG and Sorafenib

Huh‐7 cells were seeded into 96‐well plates at a density of 3000 cell well^−1^ and cultured overnight before treatment. Then, sorafenib, Gal‐LPs, Gal‐SLPs, 2‐DG, and a combination of 2‐DG and sorafenib were added into medium. The concentration of sorafenib, shUSP22 and 2‐DG was 5 µм, 0.5µg well^−1^ and 0.5 mm, respectively. Cell viability was assessed at preset timepoints (24, 48, and 72 h) by CCK‐8 assay.

##### In Vivo Pharmacokinetics Analysis

The protocol of in vivo pharmacokinetics analysis was approved by the Ethics Committee of the First Affiliated Hospital, Zhejiang University School of Medicine. Sorafenib (dissolved in Cremophor EL/ethanol (1:1)), SLP or Gal‐SLP was intravenously injected into the 6‐week‐old male ICR mice via the tail vein at a sorafenib concentration of 5 mg kg^−1^. At predetermined timepoints, blood samples (50 µL) were collected from the orbital venous plexus of mice and 1 mL of acetonitrile was added. The mixture was thoroughly vortexed, ultrasonicated, and then centrifuged at 5000 rpm for 5 min. The supernatants were taken and filtered through 0.22 µm membranes and the sorafenib content was determined by HPLC.

##### Establishment of a Sorafenib‐Insensitive Hepatocellular Carcinoma Patient‐Derived Xenograft Model

BALB/c nude mice and NOD‐SCID mice were purchased from Shanghai Experiment Animal Centre, Chinese Academy of Science. And all animal experimental protocols were approved by the Ethics Committee of the First Affiliated Hospital, Zhejiang University School of Medicine.

A schematic diagram of the establishment of the sorafenib‐insensitive HCC PDX model is illustrated in Figure 7a. Informed consent forms were signed by patients and procedures were carried out as reported previously.^[^ ^24b,c^ ^]^ In short, fresh tumor tissue was obtained from patients by surgical resection, who didn't have any chemotherapeutic or locoregional treatments before the surgery. Resected tumors were sliced into ≈1 mm^3^ wads and then subcutaneously implanted into flanks of 5‐week‐old male NOD‐SCID mice. Mice, which implanted with original patient's tumor, were defined as the Founder 0 (F0). The second generation of PDX model was defined as F1. Passage and preservation of PDX models were followed a standardized process. Owing to the fact that the PDX model, originated from patient 25 (P25), responded poorly to oral administration of sorafenib, the P25 PDX was chosen for in vivo antitumor efficiency assessment.

##### Biodistribution in Mice with HCC PDX Tumor

Near‐infrared DiR fluorescent probe was encapsulated into SLP (SLP/DiR) and Gal‐SLP (Gal‐SLP/DiR) to track the biodistribution. P25F5 PDX was established in the 5‐week‐old male BALB/c nude mice as mentioned above. When the tumor volume reached ≈200 mm^3^, the mice were intravenously administrated with SLP/DiR and Gal‐SLP/DiR at a DiR dose of 2 µg per mouse. At predetermined intervals, imaging was carried out using a NIRF imaging system (PerkinElmer IVIS Lumina XRMS Series III imaging system). At 72 h postinjection, all the mice were sacrificed. Their major organs (hearts, livers, spleens, lungs, kidneys, and intestines) and tumors were excised, washed with saline, and imaged.

##### In Vivo Antitumor Activity

In vivo antitumor efficiency evaluation was performed in the P25F5 PDX. When the tumor volume reached ≈75 mm^3^, the mice were randomized into four groups (*n* = 6). Saline, sorafenib, Gal‐LP or Gal‐SLP was intravenously injected via the tail vein every two days for a total of six injections at a sorafenib concentration of 5 mg kg^−1^ and shUSP22 concentration of 0.5 mg kg^−1^. The length and width of the tumors were recorded individually, as well as the body weights. The tumor volume (*V*) was calculated by the equation: *V* (mm^3^) = (length × width^2^)/2. At the end of the experiment, mice were sacrificed in a humanitarian way. Meanwhile, the tumor and organs were harvested for histological analysis. The tissues were fixed in 4% PFA fix solution and embedded in paraffin. The paraffin‐embedded tissues were sectioned into slices and stained with hematoxylin and eosin (H&E, Sigma). Immunohistochemistry staining of USP22 and Ki67 was executed following the manufacturer's protocols for the Immunohistochemical assay kit (Proteintech).

##### In Vivo Safety Evaluation

In vivo safety evaluation was performed in the healthy ICR mice. After intravenously injection with saline, sorafenib, Gal‐LP and Gal‐SLP for three times, the whole blood and serum were harvested and subjected for whole blood count, renal, and liver function test.

##### Statistical Analysis

Data in this study were expressed as mean ± SD of at least three independent experiments. IBM SPSS Statistics 20.0 and Prism were used for the statistical analysis. Comparison between two groups was performed using unpaired two‐sided Student's *t*‐test. One‐way ANOVA was used to compare expression of USP22, PI positive proportion, colony formation, ROS intensity, mRNA expression of USP22, and MRP1, ECAR, concentration of lactic acid, tumor volume, body weight, and tumor weight with different treatments, and post hoc test (Tukey's multiple comparisons test) was used to test difference between groups. Two‐way ANOVA plus Tukey's multiple comparisons test were utilized to test differences in the intracellular sorafenib accumulation and cell viability with different treatments for different intervals. In all cases, the differences of statistics were considered at *p < 0.05, **p < 0.01, ***p < 0.001, and ^#^ *p* > 0.05.

## Conflict of Interest

The authors declare no conflict of interest.

## Supporting information

Supporting Information
