# Supplementary material for: Self‐Activated Cascade‐Responsive Sorafenib and USP22 shRNA Co‐Delivery System for Synergetic Hepatocellular Carcinoma Therapy
Source: Adv Sci (Weinh). 2021 Jan 15;8(5):2003042. doi: 10.1002/advs.202003042 (PMC7927615; doi:10.1002/advs.202003042)
Supplement: Supplementary file 1 — Supporting Information [file ADVS-8-2003042-s001.pdf]

WILEY-VCH

© 2020 Wiley-VCH GmbH

ADVANCED SCIENCE
Open Access

# Supporting Information

for *Adv. Sci.*, DOI: 10.1002/advs.202003042

# Self-activated Cascade-responsive Sorafenib and USP22 shRNA Co-delivery System for Synergetic Hepatocellular Carcinoma Therapy

*Shengjun Xu, Sunbin Ling, Qiaonan Shan, Qianwei Ye, Qifan Zhan, Guangjiang Jiang, Jianyong Zhuo, Binhua Pan, Xue Wen, Tingting Feng, Haohao Lu, Xuyong Wei, Haiyang Xie, Shusen Zheng, Jiajia Xiang*, Youqing Shen, and Xiao Xu**

# Supporting Information

**Self-activated Cascade-responsive Sorafenib and USP22 shRNA Co-delivery System for Synergetic Hepatocellular Carcinoma Therapy**

*Shengjun Xu, Sunbin Ling, Qiaonan Shan, Qianwei Ye, Qifan Zhan, Guangjiang Jiang, Jianyong Zhuo, Binhua Pan, Xue Wen, Tingting Feng, Haohao Lu, Xuyong Wei, Haiyang Xie, Shusen Zheng, Jiajia Xiang*, Youqing Shen, and Xiao Xu**

## Supplementary Methods

*Materials and cells*: Poly[(2-acryloyl)ethyl(p-boronic acid benzyl) diethylammonium bromide] (B-PDEAEA) and fluorescein isothiocyanate (FITC)-labeled B-PDEAEA (B-PDEAEA$^{FITC}$) were synthesized as the previous literature described[1]. 1,2-Dioleoyl-sn-glycero-3-phosphoethanolamine (DOPE) was purchased from Avanti Polar Lipids Company (Alabaster, AL, USA). Cholesteryl hemisuccinate (CHEMS) was purchased from Tokyo Chemical Industry Co., Ltd (Shanghai, China). Distearoyl phosphoethanolamine–polyethylene glycol (DSPE-PEG)-galactose were obtained from Ruixi Biological Technology Co., Ltd (Xi'an, China). pGenesil-1 shUSP22 plasmids (**Figure S1**) were purchased from Vigene Biosciences Co., Ltd (Shandong, China). Plasmids were maintained and propagated in DH5α E. coli. and extracted by Plasmid Endo-Free Max Kit (Omega, USA). Sorafenib and N-acetylcysteine (NAC) was purchased from Selleck (USA).

Human HCC cell lines HuH-7 and BEL-7402 were kindly provided by Stem Cell Bank, Chinese Academy of Sciences and maintained in appropriate medium supplemented with 10% fetal bovine serum (FBS, Wisent, Canada) ,penicillin (100 mg/mL) and streptomycin (100 µg/mL) at 37 °C in humidified air containing 5% carbon dioxide.

*EGFP gene transfection efficiency*: 150000 HCC cells were plated into 6-well plates and incubated overnight. Polyplexes contained shUSP22 plasmids with different N/P ratios was added at a DNA concentration of 8 µg/well. After treatment for 24 h, cells were harvested and resuspended with PBS solution. The cells were analyzed by a flow cytometer (FACSCanto II, BD Biosciences) and the mean GFP intensity quantitatively represents the transfection efficiency of EGFP gene. For visualization of EGFP transfection, HCC cells were treated with polyplexes for 24 h and then observed with fluorescence microscope (IX71, Olympus).

*ROS scavenger assasy:* N-acetylcysteine (NAC), a typical ROS scavenger, was dissolved in DMEM medium and the pH was adjusted to 7.4 before use. For ROS scavenger assasy, Huh-7 cells were plated into 6-well plates at a density of 100000 cell/well and cultured

overnight. In NAC and NAC+Gal-SLP group, Huh-7 cells wers incubated with 10mM NAC for 2 h, followed by a 6-h incubation of PBS or Gal-SLP at a sorafenib concentration of 5 μM and shUSP22 concentration of 8 μg/dish. In Gal-SLP group, Huh-7 cells were directly incubated with Gal-SLP at a sorafenib concentration of 5 μM and shUSP22 concentration of 8 μg/dish for 6 h. After exposure, cells were stained with DHE for half an hour and subjected for flow cytometry analysis.

The influence of ROS scavenger in the Gal-SLP-induced inhibition of USP22 expression was executed via Western blot assay. Briefly, Huh-7 cells were plated into 6-well plates at a density of 100000 cell/well and cultured overnight. In NAC and NAC+Gal-SLP group, Huh-7 cells wers incubated with 10mM NAC for 2 h, followed by a 48-h incubation of PBS or Gal-SLP at a sorafenib concentration of 5 μM and shUSP22 concentration of 8 μg/dish. In Gal-SLP group, Huh-7 cells were directly incubated with Gal-SLP at a sorafenib concentration of 5 μM and shUSP22 concentration of 8 μg/dish for 48 h. After exposure, cells were harvested and Western blot assay was performed as mentioned above.

*Quantitative real-time polymerase chain reaction (qRT-PCR) assay*: Huh-7 cells were plated into 6-well plates at a density of 100000 cell/well and cultured overnight. And then cells were treated with sorafenib, Gal-LPs and Gal-SLPs at a sorafenib concentration of 5 μM and shUSP22 concentration of 8 μg/well.After incubation for 48 h, cells were harvested and isolated using RNeasy Mini Kit (QIAGEN). The cDNA was synthesized using the Hiscript II RT SuperMix for qPCR (+gDNA wiper) kit (Vazyme). Quantitative PCR was detected in ABI 7500 Real-Time PCR System (Applied Biosystems) with ChamQ SYBR Color qPCR Master Mix (Vazyme) and appropriate primers. The amount of target cDNA was analyzed through the conversion of the threshold cycle (CT). The amount of target cDNA was calculated and normalized to that of β-actin. The sequences are provided as followed. β-actin: F: CATCCACGAAACTACCTTCAACTCC; R: GAGCCGCCGATCCACACG. USP22: F:

GGACAACTGGAAGCAGAACC; R: TGAAACAGCCGAAGAAGACA. MRP1 (ABCC1): F: CTCCTCCTATAGTGGGGACATCAG; R: GTAGTCCCAGTACACGGAAAG.

*Lentiviral particle infection*: Lentiviral particles were utilized for establishing stable USP22 knockdown (SH) and overexpression (OE) in Huh-7 and BEL-7402 cells. USP22-specific shRNA and USP22 lentiviral particles were provided by Vigene Biosciences Co., Ltd (Shandong, China). $NC_{OE}$ and $NC_{SH}$ of Huh-7 and BEL-7402 were set up as control groups for USP22 OE and SH cells respectively. Procedures of lentiviral particles infection were performed as described previously[2]. The cells were further selected with 5 μg/mL puromycin (Selleck). Western blot assay was carried out to verify the successful construction of USP22 -SH and -OE cells.

**Supplementary Figures**

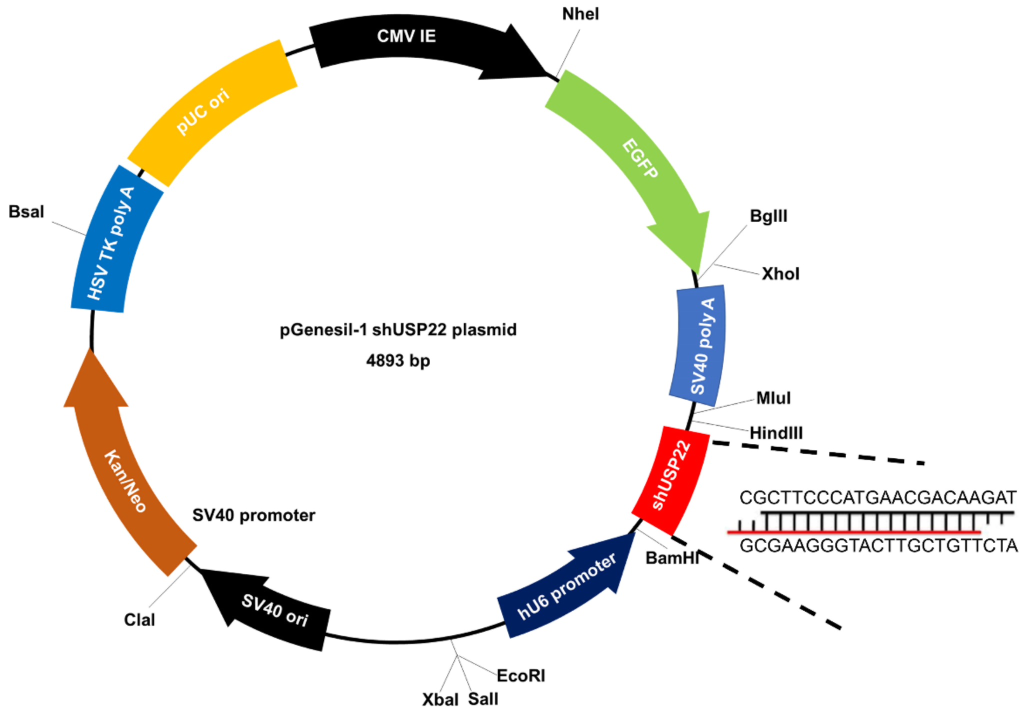


**Figure S1.** Illustrations of pGenesil-1 shUSP22 plasmid. The nucleic acid sequence of shUSP22 was reported and verified by S. Ling et al.[2], as shown in the illustration.

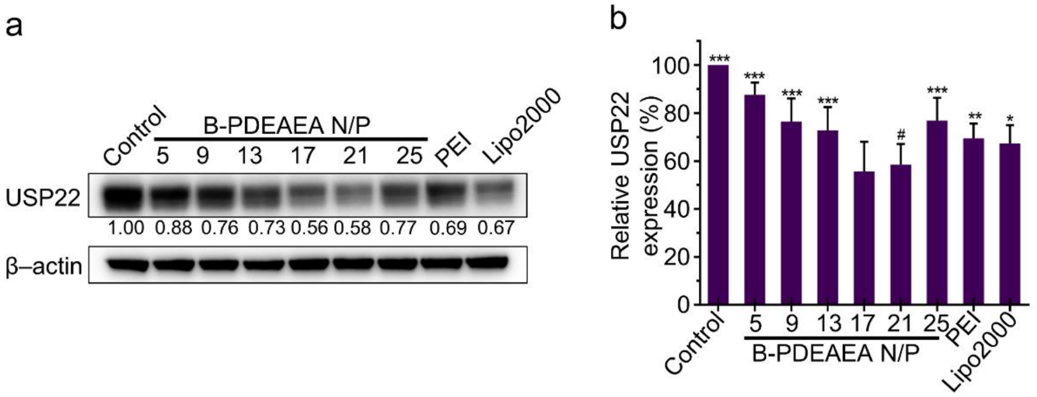


**Figure S2.** Representative Western blot analysis (a) of USP22 downregulation in BEL-7402 cells treated with polyplexes at different N/P ratios. Band intensities were semiquantified using IMAGE J software and normalized with β-actin. Means are presented under the USP22 band (a) and data are presented as the mean ± SD (n = 3) (b). PEI and Lipo2000 were used as positive controls. Statistical differences and p value between two groups (versus N/P=17) were shown as $*p<0.05$, $**p<0.01$, $***p<0.001$ and $^{\#}p>0.05$.

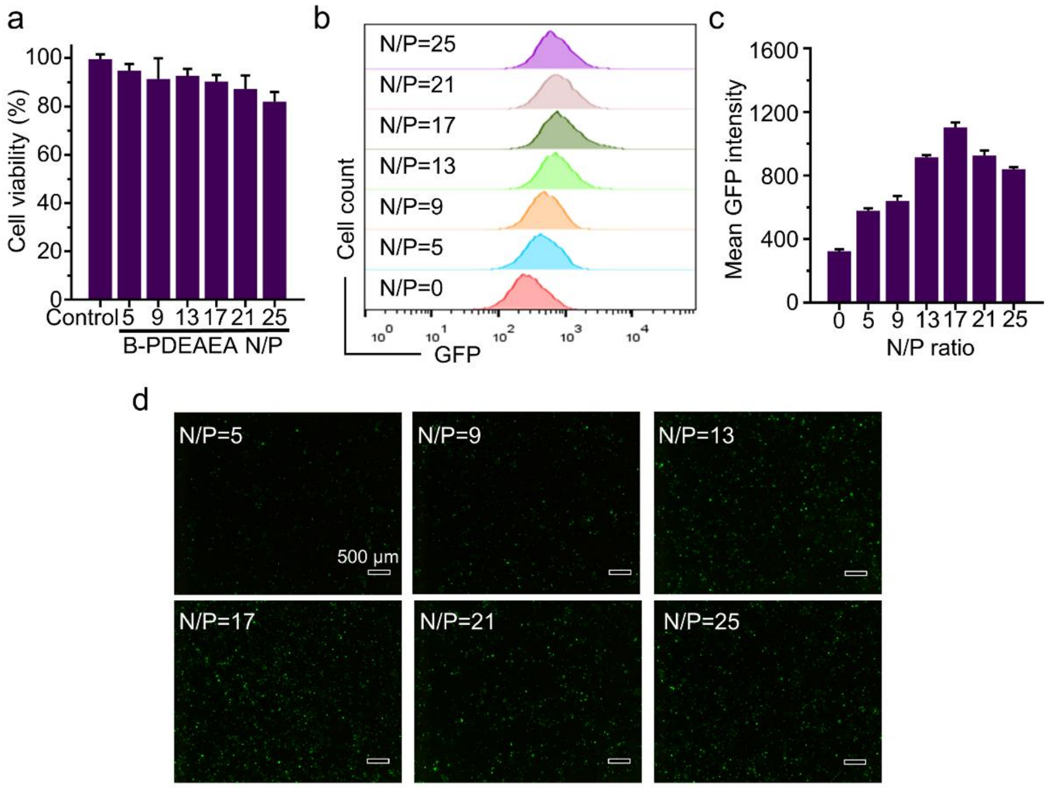


**Figure S3.** *In vitro* biosafety and EGFP gene transfection efficiency of polyplexes at different N/P ratios in Huh-7 cells. a) The cytotoxicity assay of polyplexes, containing NC plasmids, at different N/P ratios in Huh-7 cells at a NC plasmid concentration of 0.5 μg/well in 96-well plates. The data are represented as the mean ± SD (n = 4). b) Flow cytometer assay of GFP-positive Huh-7 cells treated with polyplexes for 24 h. c) Quantification of the GFP intensity of Huh-7 cells. Mean GFP intensity represents the expression of EGFP gene in Huh-7 cells. The data are represented as the mean ± SD (n = 3). d) Fluorescence observation of the transfection efficiency of EGFP gene in Huh-7 cells.

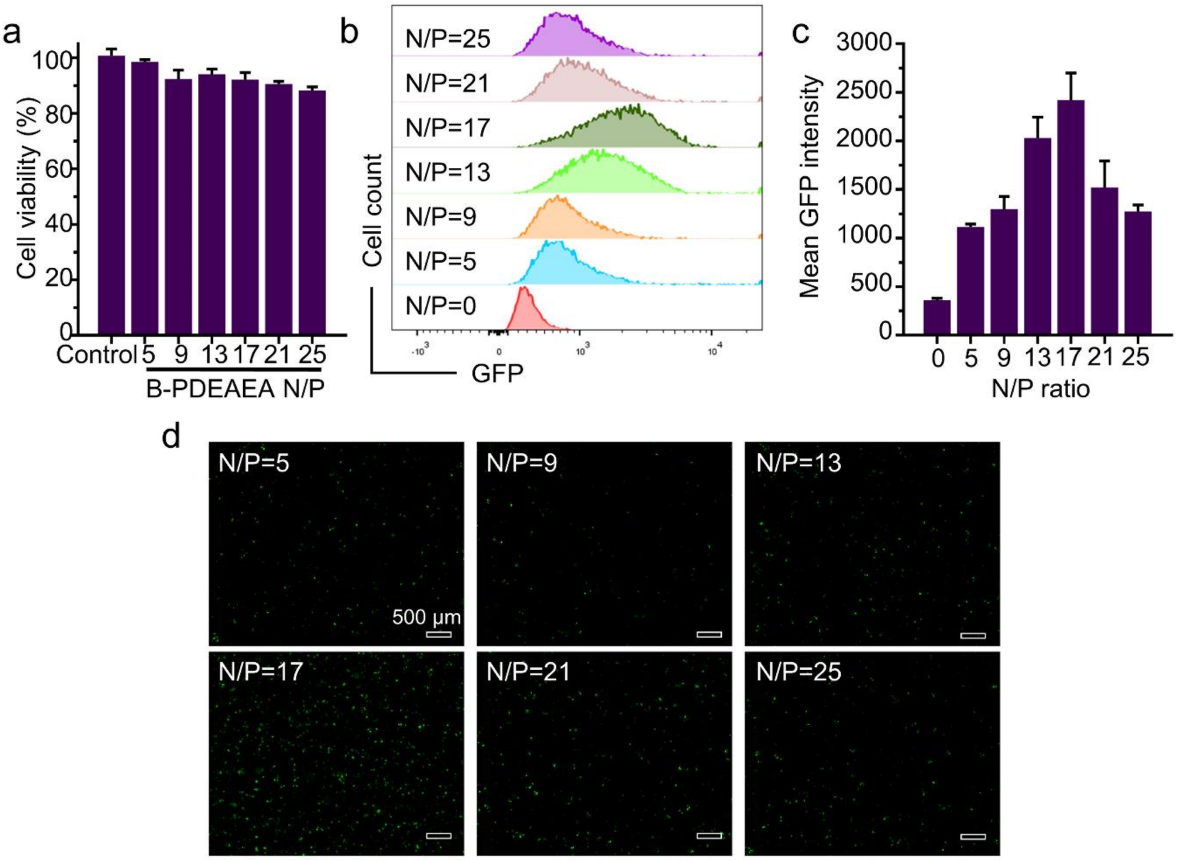


**Figure S4.** *In vitro* biosafety and gene transfection efficiency of polyplexes at different N/P ratios in BEL-7402 cells. a) The cytotoxicity assay of polyplexes, containing NC plasmids, at different N/P ratios in BEL-7402 cells at a NC plasmid concentration of 0.5 μg/well in 96-well plates. The data are represented as the mean ± SD (n = 4). b, c) Flow cytometer assay (b) and GFP intensity quantification (c) of BEL-7402 cells treated with polyplexes for 48 h. Mean GFP intensity represents the expression of EGFP gene. The data are represented as the mean ± SD (n = 3). e) Fluorescence observation of the transfection efficiency of EGFP gene in BEL-7402 cells.

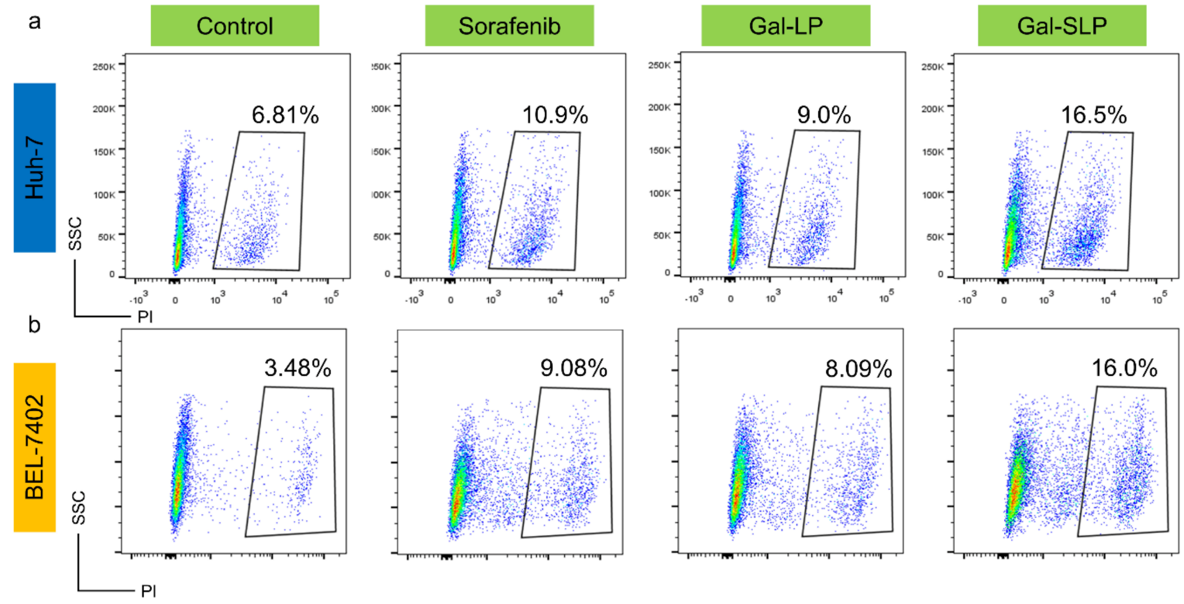


**Figure S5.** Representative flow cytometric analysis of PI staining on Huh-7 (a) and BEL-7402 cells (b) treated with PBS, sorafenib, Gal-LP and Gal-SLP at a sorafenib concentration of 5 μM and shUSP22 concentration of 5 μg/well.

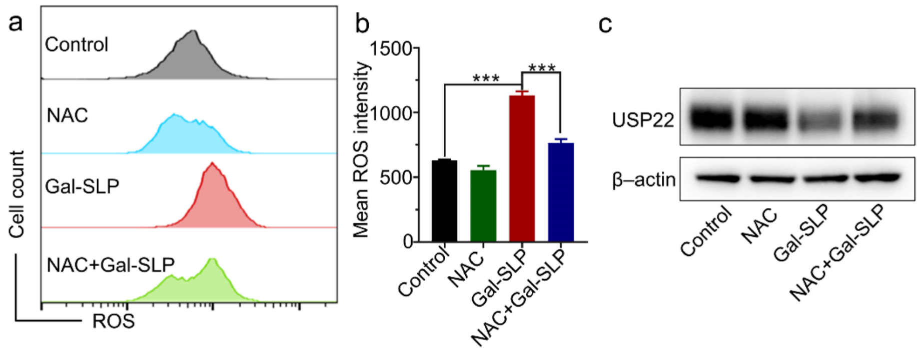


**Figure S6.** a,b) Flow cytometry analysis and quantification of intracellular ROS generation in Huh-7 cells. In NAC and NAC+Gal-SLP group, Huh-7 cells wers incubated with NAC for 2 h, followed by a 6-h incubation of PBS or Gal-SLP at a sorafenib concentration of 5 μM and shUSP22 concentration of 8 μg/dish. In Gal-SLP group, Huh-7 cells were directly incubated with Gal-SLP at a sorafenib concentration of 5 μM and shUSP22 concentration of 8 μg/dish for 6 h. The data are represented as the mean ± SD (n = 3). c) Representative Western blot analysis of USP22 downregulation in Huh-7 cells with different treatments. In NAC and NAC+Gal-SLP group, Huh-7 cells wers incubated with NAC for 2 h, followed by a 48-h incubation of PBS or Gal- SLP at a sorafenib concentration of 5 μM and shUSP22 concentration of 8 μg/dish. In Gal-SLP group, Huh-7 cells were directly incubated with Gal-SLP at a sorafenib concentration of 5 μM and shUSP22 concentration of 8 μg/dish for 24 h. *p<0.05, **p<0.01, ***p<0.001 and #p>0.05.

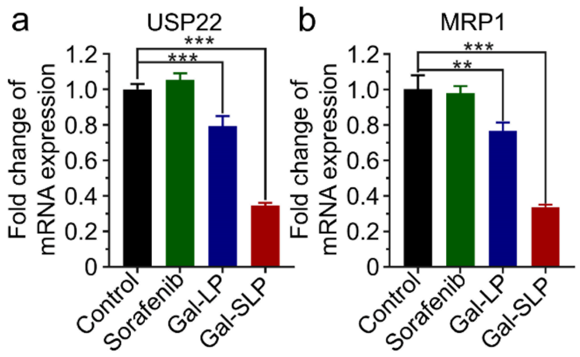


**Figure S7.** The expressions of USP22 (a) and MRP1 (b) mRNA in Huh-7 cells treated with sorafenib, Gal-LP and Gal-SLP were detected using qTR-PCR assay. The data are represented as the mean ± SD (n = 3). *p<0.05, **p<0.01, ***p<0.001 and #p>0.05.

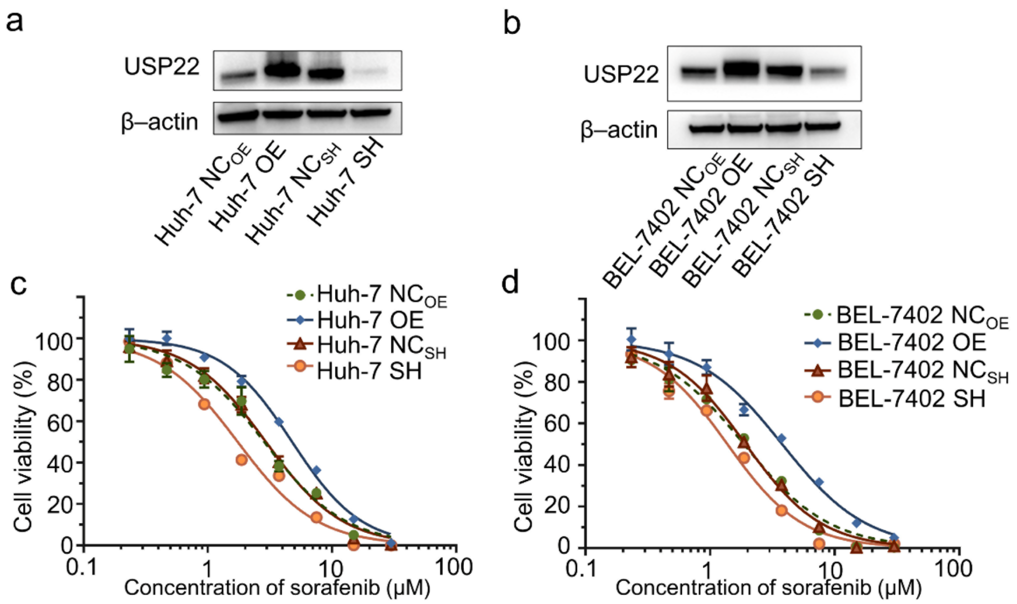


e

| USP22 expression | $IC_{50}$ (μM) of Sorafenib: Huh-7 | $IC_{50}$ (μM) of Sorafenib: BEL-7402 |
|---|---|---|
| USP22 $NC_{OE}$ | 2.943 ± 0.1086 | 1.934 ± 0.07351 |
| USP22 OE | 4.788 ± 0.1186 | 3.799 ± 0.1413 |
| USP22 $NC_{SH}$ | 2.854 ± 0.1430 | 1.874 ± 0.07905 |
| USP22 SH | 1.745 ± 0.06933 | 1.381 ± 0.04957 |

**Figure S8.** The role of USP22 in the sensitivity to sorafenib in HCC cells. a, b) The expression of USP22 in Huh-7 and BEL-7402 cells of USP22 overpression (OE), knockdown (SH) and negative controls (NC). c, d) *In vitro* cytotoxicity in HCC cells, with different USP22 expression, after 48-h treatment of different concentrations of sorafenib. The data are represented as the mean ± SD (n = 4). (e) $IC_{50}$ of sorafenib in Huh-7 and BEL-7402 cells with different USP22 expression.

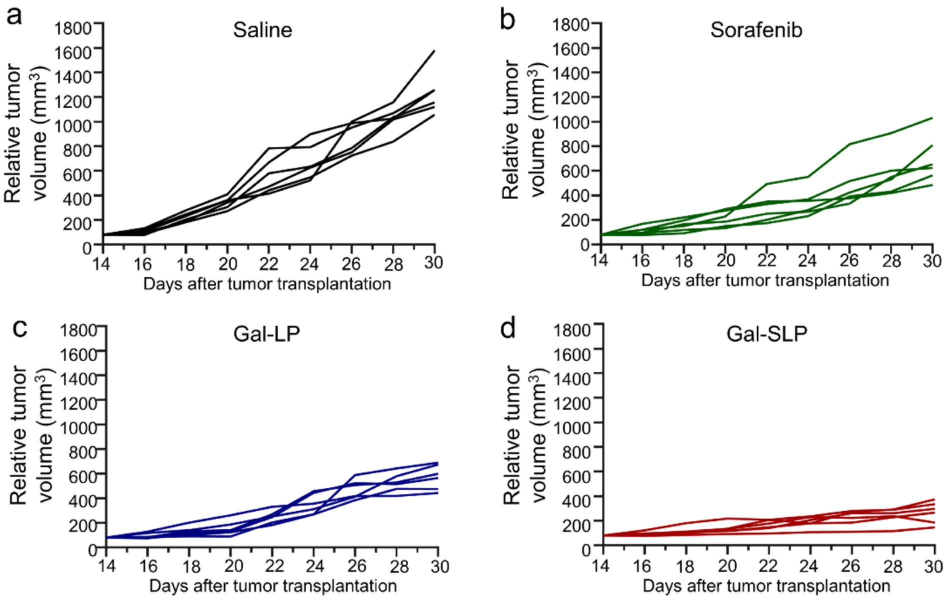


**Figure S9.** The tumor growth curves after intravenous injection of saline (a), sorafenib (b), Gal-LP (c) and Gal-SLP (d).

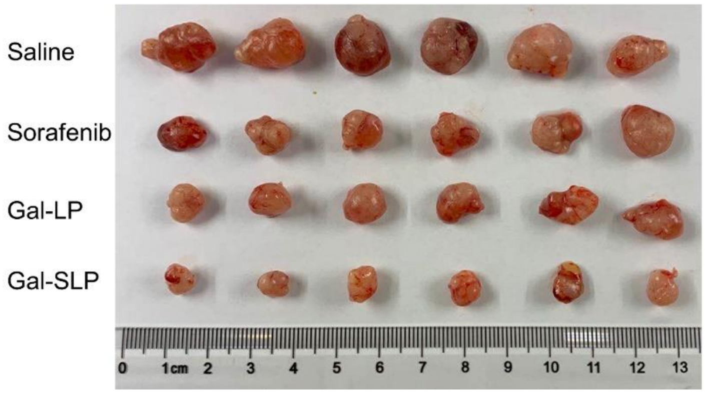


**Figure S10.** Images of the excised tumor in the different groups at the end of *in vivo* antitumor evaluation (n=6 for each group).

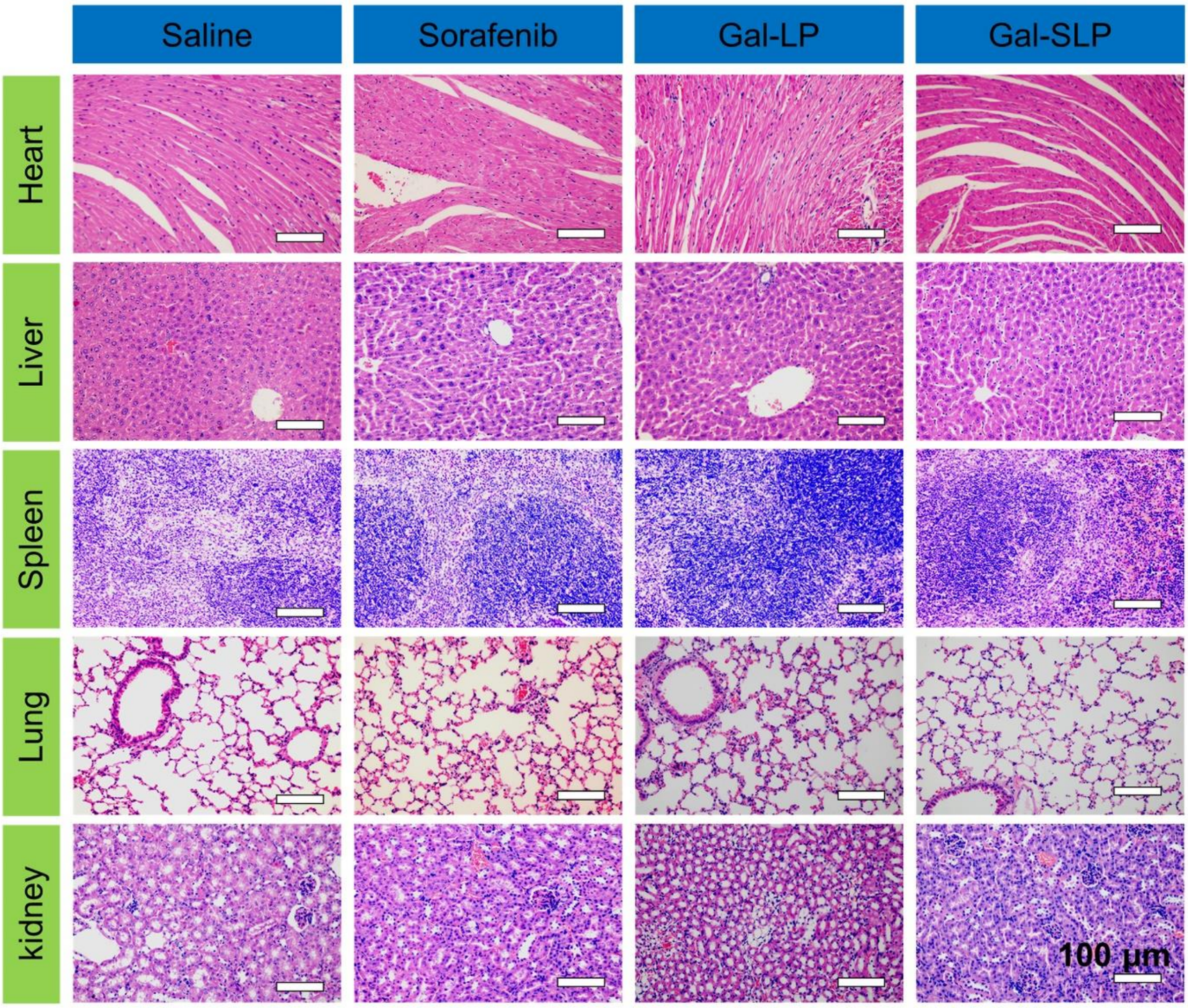


**Figure S11.** Representative Hematoxylin and eosin (H&E) staining of hearts, livers, spleens, lungs and kidneys excised from nude mice in the different groups (n=6 for each group).

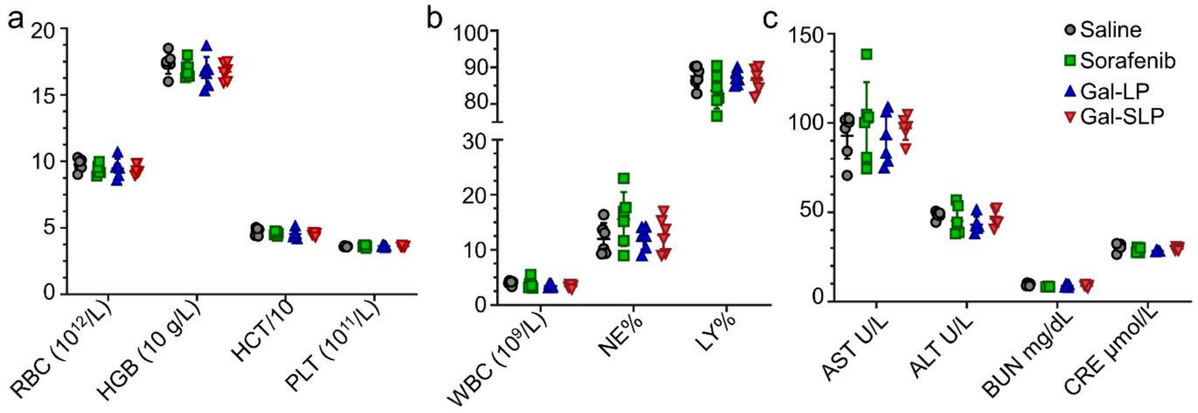


**Figure S12.** *In vivo* safety evaluation of different therapies. a, b) Whole cell counts of ICR mice treated with saline, sorafenib, Gal-LP and Gal-SLP three times. The dosages of different therapies in the biosafety evaluation was same as the dosages in *in vivo* antitumor evaluation assay. c) Liver and kidney toxicity analysis of different therapies. The data are represented as the mean ± SD (n = 6). All the indicators were within a normal biological range. RBC: red boold cell; HGB: hemoglobin; HCT: hematocrit; PLT: platelet count; WBC: white boold cell; NE%: neutrophil percentage; LY%: lymphocyte percentage; AST: aspartate amiotransferase; ALT: alanine amiotransferase; BUN: blood urea nitrogen; CRE: creatinine.

**Table S1.** Antibodies used in the Western blots and immunofluorescence staining. Without specific indications, the antibodies and their dilutions were adopted for Western bolts.

| Antibodies | Company | Product code | Dilution |
|---|---|---|---|
| Rabbit anti-USP22 | Abcam | Cat#ab195289 | 1:2000 |
| Rabbit anti-MRP1 | Servicebio | Cat#GB111041 | 1:500 (for IF) |
| Rabbit anti-AKT | Cell Signaling Technology | Cat#4685 | 1:1000 |
| Rabbit anti-pAKT (ser473) | Cell Signaling Technology | Cat#4060 | 1:1000 |
| Rabbit anti-p-GSK-3-beta (S9) | Cell Signaling Technology | Cat#5558 | 1:1000 |
| Rabbit anti-MRP1 | Thermo | Cat#PA5-30594 | 1:1000 |
| Mouse anti-beta Actin | Proteintech | Cat#60008-1-Ig | 1:10000 |
| CoraLite488 – conjugated Affinipure Goat Anti-Mouse IgG(H+L) | Proteintech | Cat#SA00013-1 | 1:200 (for IF) |
| HRP-conjugated Affinipure Goat Anti-Mouse IgG(H+L) | Proteintech | Cat#SA00001-1 | 1:10000 |
| HRP-conjugated Affinipure Goat Anti-Rabbit IgG(H+L) | Proteintech | Cat#SA00001-2 | 1:10000 |

**References**


[1] X. Liu, J. Xiang, D. Zhu, L. Jiang, Z. Zhou, J. Tang, X. Liu, Y. Huang, Y. Shen, *Advanced Materials* **2016**, 28, 1743.

[2] S. Ling, Q. Shan, Q. Zhan, Q. Ye, P. Liu, S. Xu, X. He, J. Ma, J. Xiang, G. Jiang, X. Wen, Z. Feng, Y. Wu, T. Feng, L. Xu, K. Chen, X. Zhang, R. Wei, C. Zhang, B. Cen, H. Xie, P. Song, J. Liu, S. Zheng, X. Xu, *Gut* **2020**, 69, 1322.
